# The Use of Ensemble Models for Multiple Class and Binary Class Classification for Improving Intrusion Detection Systems

**DOI:** 10.3390/s20092559

**Published:** 2020-04-30

**Authors:** Celestine Iwendi, Suleman Khan, Joseph Henry Anajemba, Mohit Mittal, Mamdouh Alenezi, Mamoun Alazab

**Affiliations:** 1 Department of Electronics, BCC of Central South University of Forestry and Tech, Changsha 410004, China; celestine.iwendi@ieee.org; 2 Department of Computer Science, Air University, Islamabad 44000, Pakistan; 130824sulemankhan@gmail.com; 3 Department of Communication Engineering, Hohai University, Changzhou 211100, China; 4 Department of Information Science and Engineering, Kyoto Sangyo University, Kyoto 603-8555, Japan; 5 College of Computer and Information Sciences, Prince Sultan University, Riyadh 12435, Saudi Arabia; malenezi@psu.edu.sa; 6 College of Engineering, IT and Environment, Charles Darwin University, Casuarina NT 0800, Australia; alazab.m@ieee.org

**Keywords:** intrusion detection system, ensemble methods, feature selection, machine learning, false positive rate, artificial intelligence

## Abstract

The pursuit to spot abnormal behaviors in and out of a network system is what led to a system known as intrusion detection systems for soft computing besides many researchers have applied machine learning around this area. Obviously, a single classifier alone in the classifications seems impossible to control network intruders. This limitation is what led us to perform dimensionality reduction by means of correlation-based feature selection approach (CFS approach) in addition to a refined ensemble model. The paper aims to improve the Intrusion Detection System (IDS) by proposing a CFS + Ensemble Classifiers (Bagging and Adaboost) which has high accuracy, high packet detection rate, and low false alarm rate. Machine Learning Ensemble Models with base classifiers (J48, Random Forest, and Reptree) were built. Binary classification, as well as Multiclass classification for KDD99 and NSLKDD datasets, was done while all the attacks were named as an anomaly and normal traffic. Class labels consisted of five major attacks, namely Denial of Service (DoS), Probe, User-to-Root (U2R), Root to Local attacks (R2L), and Normal class attacks. Results from the experiment showed that our proposed model produces 0 false alarm rate (FAR) and 99.90% detection rate (DR) for the KDD99 dataset, and 0.5% FAR and 98.60% DR for NSLKDD dataset when working with 6 and 13 selected features.

## 1. Introduction

The increase in how people view and utilize the Internet has become a blessing and also a liability to our everyday online activities. The quest for urgent data transmission on the internet and the need for commensurable security, authentication, confidentiality of web applications, and cloud interface computing have given rise to all kinds of advanced security attacks. The day to day internet usage is becoming complicated due to the threat of the internet in data security, industrial attack, and sponsored attacks to social and engineering facilities [1,2]. The complex natures of the attacks demand response with security systems that are efficient, automated, having faster responses, accuracy, and efficient security preventing systems in place.

Network intrusion detection systems (NIDS) have been developed by researchers over time that serve the purpose of detecting any suspicious action and intention that will lead to data theft or identity cloning. The fact that there has been a rapid response to security attacks on many web-based applications has not deterred the intruders from discovering loopholes to the networks and sending more sophisticated attacks.

An ExtraTrees classifier that is used in selecting applicable features for different types of Intruders with extreme learning machines (ELMs) was proposed by [1]. During attacks classification, multi-class issues were divided into multiple binary classifications and the authors used subjective extreme learning machines to solve the issue of imbalance. Lastly, they implemented in parallel the ELMs ensemble by using GPUs in order to perform in real-time intrusion detection. Their results did better than all the other methods earlier in use, achieving 98.24% and 99.76% precision on their datasets for multi-class classification. Their proposer incurred a small overhead and lacks training on how to distinguish between normal traffic and potential attacks. Meanwhile, a multi-model biomatrix recognition system that is based on pattern recognition methods was used to make a personal identification by [2]. A modification of the fingerprint was done by applying the Delaney triangulation network. Although their system achieved a high precision with low error rate equals 0.9%, it is limited and cannot function as IDS because it is based on eyelash detection and not on the internet or online system.

Another multiclass classification that uses a heterogeneous ensemble model and outlier detection in a combination of numerous approaches and ensemble methods was developed by [3]. Their study was based on Pre-processing involving a way to filter global outliers, using a synthetic minority oversampling technique (SMOTE) algorithm to repeat the sampling process. They performed a binarization on the dataset by using the OnevsOne decomposing technique. In addition, Adaboost, random subspace algorithms, and random forest were used to design their model as the base classifier. Their proposed model performed better in terms of outlier detection and classification prediction for the multiclass problem, and also did better than other classical algorithms commonly in use. The study failed to combine filtration and wrapper selection methods in order to investigate the effect of partial removal of point-outliers from datasets prior to building up of classifiers. DOS, probe, U2R, and R2L were the four types of attacks used by [4] to deal with the random forest model. They implemented ten cross-validations that were functional for classification usage and a Feature selection that was implemented on the dataset in order to reduce dimensionality, remover of redundancy and unrelated features. On comparing their random forest modeling with a j48 classier, their experimentation proves that accuracy and DR for four types of attacks are better, but they failed to use evolutionary calculation as a feature selection measure that could further improve the accuracy of the classifier. The fact is that denial of service (DoS) attacks have created massive disruptions to private and public sectors web-based applications of which many are not in the news due to management fears of customers’ panic and loss of shares. It becomes a challenge to create a multiple class-based IDS that has the capacity to withstand multiple attacks provide higher accuracy, higher detection rate (DR), and lower false detection rate (FAR).

This paper’s intention is to develop an intelligent intrusion detection system that has high accuracy, high packet detection rate, and low false alarm rate. The Objectives include 1. Developed machine learning models for the intrusion detection system; 2. Implement and evaluate the proposed solution on network security datasets; 3. Proposed a data-independent Model; 4. Achieved high accuracy; 5. Achieved high detection system; and 6. Achieved a low false alarm rate.

Our motivation is to reduce False Positive Rate (FPR) by applying dimensional reduction method on the Correlation Feature Selection (CFS) algorithm.

Our contribution includes:

1. The research performs dimensionality reduction using the Correlation-based feature selection (CFS) approach.
2. Machine Learning Ensemble Models with base classifiers (j48, Random forest and reptree) were used to perform simulations.
3. Automatically proposed optimal subset features for the new dataset.
4. FAR and Detection rate has a great impact on the IDS system, so we propose a novel solution based on machine learning ensemble models with the effect of the CFS algorithm.
5. Our Proposed CFS + Ensemble Classifiers has 0 false alarm rate and 99.90% detection rate for kdd99 dataset and for nslkdd dataset 0.5% FAR and 98.60% detection rate.
6. Our proposed model was evaluated and compared with two different datasets and also these research experimental results are also likened with other recent and important papers in this area.

The remainder of the paper is structured as stated: In Section 2, we describe the Literature review. Section 3 presented the proposed methodology. Section 4 describes the experiments and results. Section 5 concludes the research and the mindset of future work.

## 2. Literature Review

A hybrid smart system with an enhancement of the decision tree was used by the authors in [5] to design a multiple classifier system. This was done by applying Adaboost and naïve Bayes with decision trees (NBDT), non-nested generalized exemplar (NNge), and incremental pruning (JRip) rule-based classifiers (NNJR). The system was able to detect network intrusions efficiently. The only limitation to this research is that other data mining approaches were not explored in full. Hybrid IDS based on integrating the predictions of a tree by probability in a diverse kind of classifier was proposed by [6]. Their result illustrates a model that gives a much lower false alarm rate and a peak detection rate. Moreover, their proposed model shows better precision than the recent IDS models with a precision equivalent to 96.27% for KDD’99 and 89.75% for NSL-KDD—unlike authors in [7] that use spectral clustering (SC) and deep neural network (DNN) in their proposer for intrusion detection. Their results indicate that their classifier delivers a real tool of study and analysis of intrusion detection in a large network and does better than back propagation neural network (BPNN), support vector machine (SVM), random forest (RF), and Bayes tree models in spotting precision and the types of irregular attacks in the network.

The hybrid model of [8] is a proposed system designed on the network transaction that estimates the intrusion scope threshold degree at data’s peak features which are readily accessible for the physical activities. Their results show that the hybrid approach is necessary in order to achieve accuracy of 99.81% and 98.56% for the binary class and multiclass NSL-KDD datasets, respectively. Nevertheless, it was suggested for further studies to apply optimizing techniques with the intrusion detection model because it is likely to have a better accuracy rate.

A Gini index based feature selection can give the ensemble technique a higher increase accuracy of detection by 10% according to [9]. Other benefits include reduction of a false positive rate to 0.05 and improving the system performance in terms of the time it takes for executing a truer positive rate. Nevertheless, reduced features that will require less processing time in a distributed situation need to be applied to improve the detection rate.

An improved conditional variational Auto Encoder (ICVAE) with a deep neural network (DNN) was combined to design an intrusion detection model known as ICVAE-DNN by [10]. They learn and explore potential sparse representations between network data features and classes that show better overall accuracy, detection rate, and false positive rate than the nine state-of-the-art intrusion detection methods. Nonetheless, there is a need to improve the detection performance of minority attacks and unknown attacks. The adversarial learning method can be used to explore the spatial distribution of ICVAE latent variables to better reconstruct input samples. The machine learning-based IDS developed by the authors in [11] are based on deep learning. According to the authors, in large network datasets and unbalanced network traffic, the performance of the IDS may be affected, this can result in an anomaly network-based IDS. A Deep Belief Networks (DBNs) approach which projected deep learning as a swift upsurge of machine learning (ML) was proposed in [12,13]. Following this proposal, deep learning has realized greatly the extraction of high-level dormant features from dataset models. However, notwithstanding these huge successes, several problems related to IDS still exist—firstly, a high network data dimension. In many IDS models, the feature selection approach is first considered as one of the steps of the preprocessing [14]—for instance, the advancement of the Internet of Things (IoT) and the prevalent cloud-based services, in addition to the emergence of several new attacks. In the training dataset, several unidentified attacks do not appear. For instance, in the NSL-KDD dataset considered in [15,16], about 16.6% of the attack samples in the dataset tested did not appear in the training dataset. This implies that mostly all conventional IDS typically achieve poor performance. However, for an anomaly network-based IDS (A-NIDS), the authors in [17,18] proposed a primal dependable hybrid approach that incorporates the Adaboost meta-algorithm and artificial bee colony (ABC). This is intended to achieve optimal detection rate (DR) at a minimized false positive rate (FPR) [19]. In the study by [20], the ABC algorithm is implemented for selection of features, while the Adaboost meta-algorithm is used for feature classification and evaluation. The Adaboost meta-algorithm was implemented to tackle the unbalanced data based on the actual plan, while the ABC was used for the IDS problem optimization. Incorporating both the redesigned density peak clustering algorithm (MDPCA) and the deep belief networks (DBNs) resulted in a novel fuzzy aggregation approach which was proposed in [21]. The MDPCA section of the algorithm splits the primal training dataset into numerous minor subsets based on the similarity of the training samples feature. On the other hand, the results of the entire sub-DBNs classifiers are combined according to the weights of the fuzzy membership. The objective of [22] was to design a system that has to have the capacity for accurate traffic classification of classes into normal and attack, measure up the huge datasets, and be able to acquire a lower false alarms rate. To achieve these, the authors leveraged on the Extreme Learning Machine (ELM) algorithm, which is an advanced ML algorithm. Although the ELM algorithm has proved to be more efficient in terms of performance against the Support Vector Machine (SVM) algorithm, it operates, however, at high frequency while sustaining adequate classification ability. The authors further attempted to enhance the performance ELM algorithm by including a redesigned kind of Huang’s Kernel-based ELM and combined this with the Multiple Kernel Boost (MKBoost) framework which was earlier introduced by [3]. A novel approach based on the combination of discretization, filtering, and classification methods using a KDD Cup 99 dataset is presented in [23]. The focus of the research was to drastically minimize the number of features while classifier performance is absolutely maintained, or even improved. The approach makes use of filters because of their high-speed characteristics and based on their high suitability for large datasets. Deep learning models were applied as classifiers. Bearing in mind the importance of the temporary data classification of network attacks, the Long Short Term Memory (LSTM) network, a modification of frequent networks, was used in classifying the KDD’s dataset attacks [24]. Several works in the literature of [25,26] motivated the development of our proposed approach. A scheme of nested binary trees was used in [26]; the scheme realized a good performance when tested with minor UCI datasets, but the computational difficulty of this scheme amplified swiftly with the increase at the number of instances. The recent study of [25] integrated both the oversampling and binarization with boosting, and indicated that the proposed approach realized improved performance than the multiclass learners and one-versus-all (OVA) framework. Even though information about the runtime was voided in the study, the use of oversampling enhances substantial computational difficulty; hence, this method failed to scale proficiently for an application to IDS datasets, which encompasses a higher number of samples. On the other hand, the authors in [26] implemented random undersampling (RUS) in their method because it can realize similar performance when used for all the datasets while dealing with class imbalance mitigation.

Several studies on the use of binary classifiers set to the detection of intrusion have been established. A good number of these studies engaged the use of classifiers based on SVM. Authors presented a simple decision tree–based OVA model which populates a decision tree structure using a set of class probabilities [27]. An OVA method in [28] was also incorporated into a least-squares SVM technique and analyzed on the KDD dataset. The output showed that, for each of the five classes of traffic, their attack detection rate was approximately 99%. Additionally, the authors observed in the method, the best model realized an average FPR of 0.28%. SVMs in a binary classification method was employed by [29]. Authors in [30] proposed a composite scheme architecture in which precise classifiers were allocated the task of detecting precise classes. For example, an SVM was allocated for the detection of DoS attacks, while an RBF-based neural network was allocated for the detection of U2R-based attacks. The results of the hybrid classifier were transferred to a different ensemble which was allocated for the detection of R2L and probe attacks. For this scenario, in advance, a definite architecture was defined. A weighting element was included in a scheme of binary SVMs in [31]. The binarization methods that were tested included one-versus-one (OVO), OVA, directed acyclic graphs, and ECOC. It was noticed that the OVA model distributes the best performance. It is observed by the authors that the weight which measures a prediction level of certainty was targeted at the unclassifiable areas in which the group of binary classifiers cannot approve on a single class prediction. using a precise subset of the KDDTest+ dataset, the model was assessed, but then the outputs proved that employing a weighting system with the model resulted in an improved general performance better than the model that did not include weighting scheme. Individual class performance on binarization approaches have been analyzed in all the above-mentioned works; however, the lowest FPR was realized in the recent works [32,33,34,35,36] while many other algorithm and DoS were considered by [37,38,39,40,41,42,43,44].

## 3. Proposed Methodology

**Figure 1:**
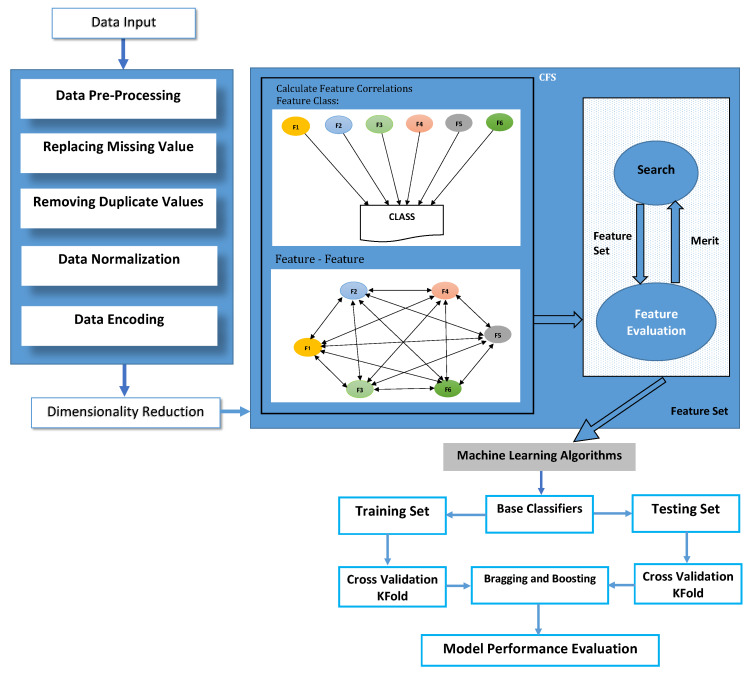
Proposed methodology.

This research has five phases according to our proposed methodology shown in Figure 1; the 1st phase is data collection. After data collection, the next phase is data pre-processing, which is phase 2. In data pre-processing, duplicate values inside the dataset are removed. Inconsistent values are also removed. Missing values were checked for its presence or not in the dataset. Data normalization was also done to bring down the whole dataset into one standard scale. Non-numeric values were converted to numeric by doing encoding. After data pre-processing, the 3rd phase is dimensionality reduction, which was done by using the CFS method. After dimensionality reduction, the next phase, which is the 4th phase, comes in the 4th phase machine learning ensemble classifiers Bagging, and Adaboost was used. The 5th phase is an evaluation phase; in this phase, this research work is compared with other state-of-the-art work that used the same approach.

### 3.1. Description

This research uses two datasets: the KDD99 dataset and the NSLKDD dataset.

#### 3.1.1. KDD99 Dataset

KDD99 is one of the most famous and old data sets used in network security for intrusion detection systems. KDD99 is a derived version of the 1998 DARPA. The kdd99 dataset was developed in an MIT research lab, and it is used by IDS designers as a benchmark to evaluate various methodologies and techniques [40]. The kdd99 has 4,900,000 rows and 41 attributes, and one is class label. Twenty-two network attacks are listed in the KDD99 dataset [41]. In this research, we did binary classification as well as multiclass classification for kdd99 and nslkdd datasets. We named all the attacks as an anomaly and normal traffic and then performed experiments. Class labels consist of four major attacks like DoS, Probe, U2R, R2L, and Normal class. We did further classification in DoS, Probe, U2R, and R2L, in order to detect the categories of these attacks.

Table 1 represents the total number of normal and anomaly packets that contain the KDD99 dataset used in this research. 97,277 and 396,731 packets were used for anomaly and normal classes to develop ensemble machine learning classifiers upon which training and testing can be performed. In addition, 70% of the KDD99 dataset was used for training and validation purposes, and the rest of the 30% dataset was used for testing and validation, respectively. The samples for KDD99 Training and Testing are present in Table 2.

Table 3 represents the number of attacks used in this research for prediction and their number of packets (size). DoS has five sub-attacks in it. The similarity Probe and R2L have four sub-attacks in it, respectively.

#### 3.1.2. NSLKDD Dataset

NSLKDD is a derived version of the KDD99 dataset. NSLKDD does not have any duplicate values that were in the kdd99 dataset. NSLKDD also does not have inconsistent values. NSL-KDD contains 148,517 instances for training and testing purposes overall. The NSLKDD set has 41 features in total. Some features are binary, some are numeric, and nominal features are also listed in the NSLKDD dataset. The NSLKDD dataset also consists of four major attacks like DoS, Probe, U2R, R2L, and Normal class.

Table 4 represents the total number of normal and anomaly packets containing the NSLKDD dataset used in this research. The total number of anomaly and normal packets used to train and test machine learning ensemble models are 71,215 and 77,054, respectively. In addition, 70% of the NSLKDD dataset was used for training and rest of the 30% dataset was used for testing and validation, respectively.

Table 5 represents the total number of anomaly and normal packets used to train and test machine learning ensemble models are 103,789 and 44,481, respectively. The number of attacks for NSLKDD and Features of KDD99 and NSSLKDD datasets are presented in Table 6 and Table 7, respectively.

### 3.2. Pre-Processing

#### 3.2.1. Normalization

After selection of the dataset, data cleaning operations are performed on datasets to remove noise from the dataset and normalize the features. For normalization, different techniques are used, but, in this research, the min-max normalization approach is used which is better in terms of scaling and solving outliers’ issues with z-score normalization. Min-max scaling normalizes values in the range of [0, 1]. The equation for min-max normalization is given below:

(1) $$Z_{i}=\frac{Y_{i}-\min\left(Y\right)}{\max(Y)-\min(Y)}$$

$Y=(Y_{1},Y_{2},Y_{3},\ldots ,Y_{n})$ are the number of features, while *Y_i_* is the feature that we want to normalize and Zi is the normalized feature. By doing this, now all features have the same weights and all features are in one scope.

#### 3.2.2. Data Encoding

In the process of data encoding, duplicate and inconsistent values were removed earlier from the datasets before the commencement of this process. The next process was to convert the nominal attributes to numeric values. The reason for this is that machine learning algorithms’ back-end calculations are done using numeric values and not nominal values. This data encoding step is vital before we proceed to passing data to the proposed model.

### 3.3. Feature Selection

Optimal features not only improve accuracy, but also improve computational cost in terms of time. The main focus of feature optimization is not only to decrease the computational cost but also find such feature subsets that can work with different classifiers to produce better results. In this research, we used the correlation-based feature selection method (CFS) for feature selection.

#### Correlation-Based Feature Selection (CFS)

**Figure 2:**
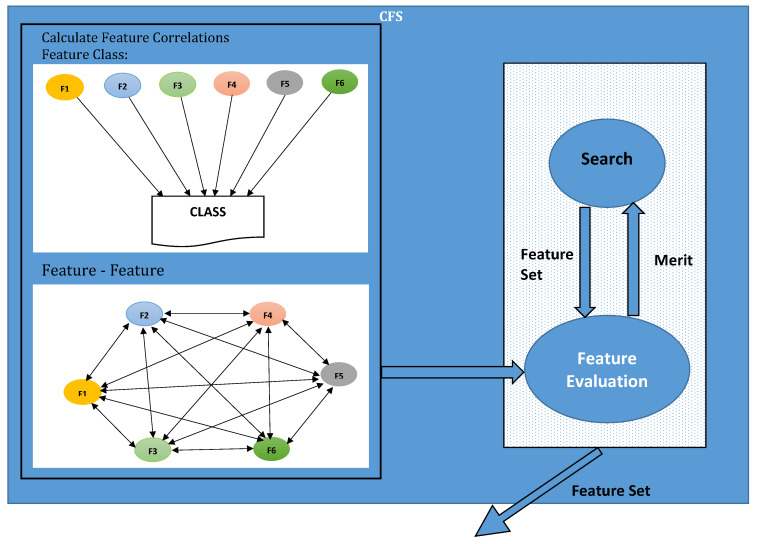
CFS work flow.

Figure 2 illustrates the workflow of the TCFS model. In the illustration, feature selection algorithms not only reduce dimensionality, but also select optimal features that produce high results in terms of accuracy, precision, recall, and F1-Scores. Dimensionality reduction also decreases the computational cost of algorithms. Heuristic evaluation function is used inside the Correlation-based feature selection (CFS) algorithm, which is dimensionality reeducation algorithm [45,46,47]. CFS ranks features based on their similarity with the predication class. CFS examines every feature vector subset. These subsets of feature vectors are highly correlated with the predication class but irrespective of each other. The CFS algorithm considers that some features have a low correlation with the predication class, so these features can be ignored because these features have no major role in prediction. On the other side, it is important to evaluate excess features since they are generally strongly associated with each other or with other features as well. The following equation can be used to find a subset of features vectors correlated with each other:

(2) $$M_{s}=\frac{A\bar{M_{cf}}}{\sqrt{A+A\left(A-1\right)M_{ff}}}$$

If we have *S* number of features subset having *A* number of attributes, then $M_{s}$ is evaluation of these *S* subsets with *A* number of attributes, where ${\bar{M}}_{cf}$ represents the average correlation between class label and attributes. $M_{ff}$ is average correlation values between attributes, or we can say how much two features are associated with each other based on this $M_{ff}$ function [37]. If we have a classification problem, CFS calculates symmatrix uncertainty shown in Equation (3):

(3) $$SU=\left[\frac{E\left(X\right)-E\left(\frac{X}{Y}\right)}{E\left(X\right)+E\left(\frac{X}{Y}\right)}\right]$$

In Equation (3), *E* represents the entropy function that is calculated using below Equation (4). Entropy is a function of the uncertainty of a random variable:

(4) $$E\left(X\right)=-\sum_{y\in X}p\left(y\right){log}_{2}p\left(y\right)$$

(5) $$E\left(\frac{X}{Y}\right)=-\sum_{w\in Y}p\left(w\right)\sum_{w\in X}p\left(\frac{y}{w}\right){log}_{2}p\left(\frac{y}{w}\right)$$

For all values of *X*, *P (y)* represents the prior probabilities while, when *Y* given to *X*, *P (y/w)* is the posterior probability.

Six features were selected using the KDD99 dataset for binary class and 11 features were selected for 21 attacks for the KDD99 dataset. Similarly, for the nslkdd dataset, 13 features were selected for both binary and multiple attacks as shown in Table 8. The correlation-based feature selection working algorithm which describes the modalities of the CFS model is presented below as Algorithm 1.

**Algorithm 1:** Correlation-based feature selection (CFS) working algorithm.**Input of data:** S (A1, A2, A3, A4, C) // Clean dataset δ// Benchmark threshold value **Output:**
$S_{opt}$ Optimal Features vector 
StartFor I = 1 to N do startMeasure $SU_{i,c}$ for every attribute $A_{i}$;If ($SU_{i,c}\geq \delta$)Then, Append $A_{i}$ to list $S_{list}^{{}^{\prime }}$;end

### 3.4. Bagging Classifier

An ensemble method is a technique that combines the predictions from multiple machine learning algorithms together to make predictions more reliable than any other (see Algorithm 2). Bootstrapping or Bagging is a very effective and powerful ensemble approach. In ensembling, multiple classifiers are combined to get more accurate results, compared to their individual performance. Working on Bagging is given below in the form of a pseudo code.

**Algorithm 2:** Bagging classifier algorithm.**Input:** KDD99 and NSLKDD datasets **Training:**
Selection of the number of samples for Bagging which is n samples and also the selection of base classifier C (j48, Random Forest, and reptree in our case).Dividing dataset into two subsets (Training and Testing subsets). Produce further training datasets using with replacement sampling and these datasets are $D_{1}D_{2}D_{3}.........D_{n}$.Then, train a base classifier on each dataset $D_{i}$ and build n number of classifiers $C_{1}C_{2}C_{3}.........C_{n}$.
**Testing:**
In the testing dataset, each data object X is passed to trained classifiers $C_{1}C_{2}C_{3}.........C_{n}$.The label is assigned to every new data object based on a majority vote. For the classification problem, the majority vote is used to assign a new label to data point X and, for the regression problem, the average value is used to be assigned to a new data object $X_{i}$.We repeat these steps until we classify every object in the dataset.

### 3.5. Adaboost Classifier

The goal of the Adaboost classifier is converting weak classifiers into a strong classifier that produces better results:

(6) $$H\left(X\right)=sign\left(\sum_{n=1}^{N}\theta_{n}h_{n}\left(x\right)\right)$$

$h_{n}$ represents the *n*th weak classifier and $\theta_{n}$ is the corresponding weight for that classifier. The Adaboost Classifer is given in Algorithm 3.

**Algorithm 3:** Adaboost classifier algorithm**Input:** KDD99 and nslkdd datasets **Training:**
Selection of base classifier C;Set the threshold for initial weights $W_{i1}\in$ [0, 1], ${sum}_{i=1}^{N}$$w_{i1}$ = 1, Commonly $W_{i1}=\frac{1}{N}$;For n = 1 →*k produce sample* $D_{n}$ for training from D using the distribution $W_{n}$Training of base classifier C on $D_{n}$ data subset to develop the $C_{n}$ classifier.$e_{n}=\sum_{j=1}^{N}w_{ni}$ is the ensemble error calculated when classifier $C_{n}$ misclassifies the $i^{th}$ data point in D.If $e_{n}\in$ (0, 0.5) then calculate $\beta_{n}=\frac{e_{n}}{1-e_{n}}$ and update the next weight.$w_{n+1,i}=\left\{\begin{array}{c} w_{ni}X\beta \\ w_{ni} \end{array}\right\}$Distribution $W_{n+1,i}$ needs to be normalized.For further value of $e_{n}$, set threshold $W_{i1}=\frac{1}{N}$ and continue the process;Return the trained classifiers $C_{1}C_{2}C_{3}.........C_{n}$ and $\beta_{1}\beta_{2}\beta_{3}......\beta_{n}$.
**Testing:**
In the testing dataset, each data object X is passed to the testing dataset; classify by classifiers $C_{1}C_{2}C_{3}.........C_{n}$.For each label y, assign to x by $C_{n}$, calculate $m_{y}\left(x\right)=\sum C_{n}\left(x\right)=yln\left(\frac{1}{\beta_{k}}\right)$. The class that has maximum value $m_{y}\left(x\right)$ is decided as the class label of x.Repeat step2 for testing data and return the output.

### 3.6. Evaluation Matrixs

Various performance matrixs are used to evaluate the proposed solution, including precision, recall, F1-Measure [48], False Alarm Rate (FAR), Detection Rate (DR), and Accuracy. The above-mentioned performance matrixs are based on True Positive (TP), False Positive (FP), False Negative (FN), and True Negative (TN).

False Positive Rate is a combination of total instances that are normal but classified as attack class and truly classify attack class.

(7) $$FalsePositiveRate\left(FPR\right)=\frac{F_{p}}{F_{p}+T_{n}}$$

Accuracy is used to measure how many instances are correctly classified as normal and attack classes. Accuracy is achieved by summing correctly classify instances and dividing the total instances as shown in Equation (8):

(8) $$Accuracy=\frac{T_{p}+T_{n}}{T_{p}+F_{p}+F_{n}+T_{n}}$$

Detection Rate (DR) provides information about the attacks detected correctly divided by the total number of attacks in the dataset:

(9) $$TruePositive=\frac{T_{p}}{T_{p}+F_{n}}$$

Precision’s objective is to evaluate the True Positive (TP) entities in relation to False Positive (FP) entities:

(10) $$Precision=\frac{T_{p}}{T_{p}+F_{p}}$$

The purpose of recall is to evaluate True Positive (TP) entities in relation to (FN) False Negative entities that are not at all categorized. The mathematical form of recall is mentioned in Equation (10):

(11) $$Recall=\frac{T_{p}}{T_{p}+F_{n}}$$

Sometimes, performance assessment may not be good with accuracy and recall. For instance, if one mining algorithm has low recall but high precision, then another algorithm is needed. Then, there is the question of which algorithm is better. This problem is solved by using an F1-Score that gives an average recall and precision. F1-Score can be calculated as shown in Equation (11):

(12) $$F1-Score=\frac{2\;*\;\mathrm{Precision}\;*\;\mathrm{Recall}}{\mathrm{Precision}+\mathrm{Recall}}$$

## 4. Experiments

The simulation was performed using Weka 3.7 [49] on Intel^®^ Core™ i3-4010 CPU@1.70 Ghz (4 CPUs) with 8 GB RAM installed. Haier laptop was used with a 64-bit operating system on it. In this research, for experiments, two datasets were used: KDD99 [50] and nslkdd [51]. The KDD99 dataset is an advanced version of the DARPA 1998 dataset. The main feature that separates the KDD99 dataset from DARPA 1998 is that test data are not from the same probability distribution as training data; it has different attacks for testing that training data doesn’t have. Similarly, NSLKDD are an advanced version of the KDD99 dataset. The NSLKDD dataset solves the problem of duplicate and inconsistent values that the KDD99 dataset had.

### 4.1. Binary Class Experiment Results for KDD99

Table 9 depicts that false positive rate and true positive rate scores were 0.6% and 99.10%, respectively, for a normal class. Similarly, for the anomaly class, false positive and true positive scores were 0.9 and 99.40%, respectively. For normal class, the number of correctly detected packets was 28,934, and 271 packets were detected incorrectly as anomaly packets. In addition, for the anomaly class, 118,238 packets were correctly detected while 759 packets were incorrectly detected as normal packets. From Table 10, we can see that precision for normal was class 97.40%, Recall score for normal class was 99.10%, and F1-Score was 98.30%, respectively. Likewise, for anomaly class, Precision and Recall scores were 99.80% and 99.40%, respectively. F1-Score for anomaly class was 99.60%. The ROC Area for both normal and anomaly class were 99.90%, respectively.

Table 11 depicts that, for normal class, the number of correctly detected packets is 28,934, and 271 packets were detected incorrectly as anomaly packets. Similarly, for anomaly class, 118,238 packets were correctly detected, while 759 packets were incorrectly detected as normal packets. From Table 12, we can see that precision for normal was class 97.40%, Recall score for normal class is 99.10%, and F1-Score was 98.30%, respectively. Similarly, for anomaly class, Precision and Recall scores were 99.80% and 99.40%, respectively. F1-Score for anomaly class was 99.60%. The ROC Area for both normal and anomaly class was 99.90%, respectively.

Table 13 indicate that, out of 148,202 instances, 147,314 instances were classified correctly with the accuracy of 99.80%. False Positive Rate and True Positive Rate score were 0.6% and 99.20%, respectively, for a normal class. Similarly, for anomaly class, False Positive and True Positive score were 0.98 and 99.40%, respectively. For normal class, the number of correctly detected packets were 28,975, and 230 packets were detected incorrectly as anomaly packets. Likewise, for the anomaly class, 118,339 packets were correctly detected, while 658 packets were incorrectly detected as normal packets. From Table 14, precision for normal class is 97.80%, recall score for normal class is 99.20%, and F1-Score was 98.50%, respectively. In addition, for anomaly class precision and recall scores were 99.80% and 99.40%, respectively. F1-score for anomaly class was 99.60%. The ROC Area for both normal and anomaly class was 99.80% and 100%, respectively.

As shown in Table 15, correctly detected normal and anomaly packets were 28,838 and 118,225, respectively. In addition, 367 packets were wrongly classified as anomaly, but, actually, it was normal packets. Similarly, 772 packets were anomaly, but it was detected as normal packets.

According to Table 16, using the Bagging j48 classifier, the false positive rate and true positive rate scores are 0.6% and 98.70%, respectively, for a normal class. Similarly, for the anomaly class, false positive and true Positive scores were 1.30% and 99.40%, respectively, using a j48 classifier.

Table 17 depicts a Bagging random forest classifier detects 28,994 packets correctly as normal packets and 118,318 packets as anomaly packets. In addition, 211 packets are detected as anomaly packets, but, actually, they are normal packets and 679 packets were detected as normal packets, but, actually, they were anomaly packets.

For Bagging random forest classifier precision, recall, and F1- score for the normal class are 97.70%, 99.30%, and 98.50%, respectively. Similarly, for Bagging random forest, anomaly class precision is 99.80%, the recall is 99.40%, and F1-score is 99.60%, respectively. using Bagging random forest normal class, False Positive Rate was 0.60% and, for anomaly, False Positive Rate was 0.90%. True Positive score for Bagging random forest normal class was 99.10% and, for anomaly, 99.40%, respectively, as shown in Table 18

As shown in Table 19, correctly detected normal and anomaly packets are 29,010 and 118,299, respectively. In addition, 195 packets are wrongly classified as anomaly, but, actually, they are normal packets. Similarly, 698 packets were an anomaly, but they are detected as normal packets. False Positive and True positive scores for normal are 0.60% and 99.30%, respectively. Similarly, for anomaly class, False Positive and True positive scores are 0.70% and 99.40%, respectively, as shown in Table 20. For reptree Bagging normal class, precision score is 97.70%; recall and F1-Scores are 99.30% and 98.50%, respectively. For Bagging reptree, anomaly class precision score is 99.80%, recall score is 99.40%, and F1-Score was 99.60%, respectively, as shown in Table 20.

**Figure 3:**
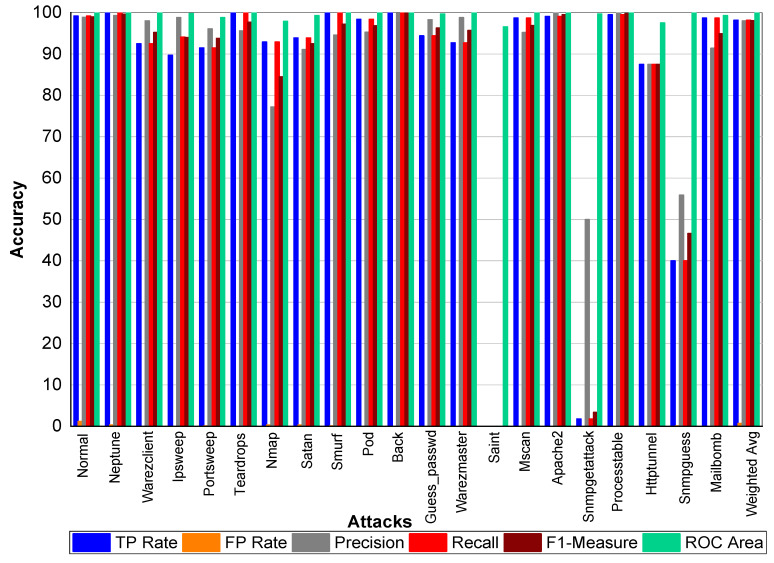
Classification report for the Adaboost J48 KDD99 dataset.

Table 21 and Figure 3 indicate that Perl, Neptune, Smurf, Guess_passwd, Pod, Teardrop, and Lad attacks have 100% TP Rate. Only three attacks Loadmodule, Ftp_write, Phf have a very low TP Rate. The weighted average TP Rate is 99.90 overall. The FP Rate for all attacks are very low. Normal packets achieve 99.80% precision, Loadmodule, Neptune, Smurf, Teardrop, Portsweep, Imap, and Warezmaster achieved 100% precision, respectively. Guess_passwd achieved 93.80% precision, and Portsweep achieved 95.30% precision. Ipsweep and Land achieved 81.60% and 83.30% precision, respectively. Perl and Multihop achieved 33.33% precision, respectively. Back, Satan, and Warezclient achieved 99.80%, 99.10%, and 97.10% Precision, respectively. Perl, Neptune, Smurf, Guess_passwd, and Pod achieved 100% recall. Teardrop and Land also achieved 100% recall, respectively. Normal, Guess_passwd, Portsweep, Ipsweep, Back, Satan, and Warezclient achieved more than 90% F1-score on average. Neptune, Smurf, Pod, and Teardrop achieved a 100% F1-score, respectively. Buffer overflow, Loadmodule, Ipsweep, Nmap, and Warezclient achieved more than 99% average ROC. Multihop and Warezclient achieved 81.70% and 71.70% ROC, respectively. All other attacks achieved 100% ROC, respectively.

**Figure 4:**
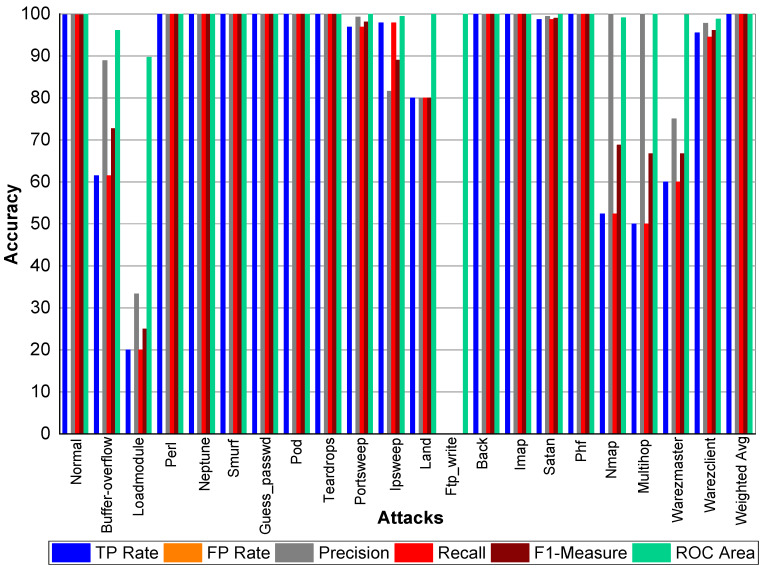
Classification report for Adaboost Random Forest KDD99 dataset.

TP and FP Rate for normal class are 99.8% and 0%, respectively. Precision, recall, and F1-score for a normal class was 99.90%. Similarly, Perl, Neptune, Smurf, Guess_passwd, Pod, Teardrop, Back, Imap, and Phf achieved 100% precision, recall, F1-score, TP Rate, and ROC area, respectively. Buffer_overflow achieved 61.50% TP Rate, 88.90% precision, 61.50% recall, 72.70% F1-Measure, and 96.10% ROC area. Loadmodule attack achieved a 20% FP Rate and 20% recall. Precision and F1-Measure for Loadmodule were 33.33% and 25.00%, respectively. Portsweep achieved a 96.90% FP Rate and recall, respectively. Precision and F1-Measure for Portsweep were 99.30% and 98.10%, respectively. Warezclient, Warezmaster, Multihop, Nmap, and Satan also performed very well in terms of precision, recall, and F1-Measure—as shown in Table 22 and Figure 4.

**Figure 5:**
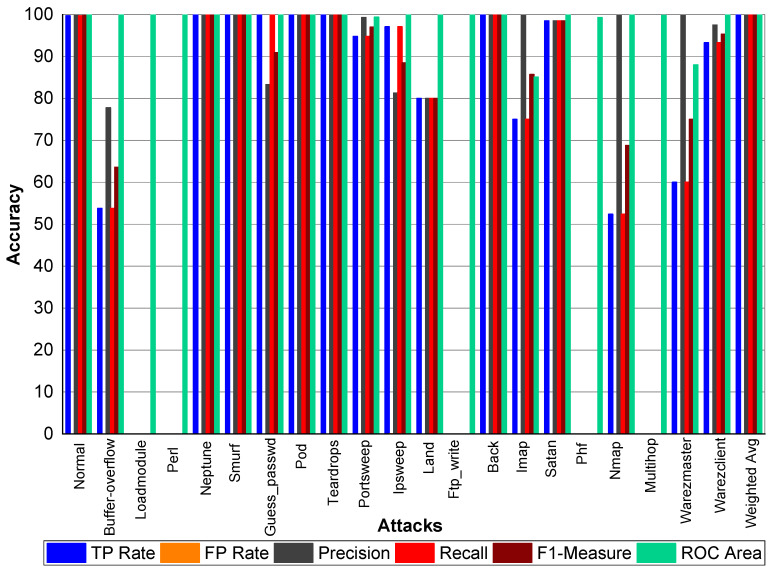
Classification report for the Adaboost Reptree KDD99 dataset.

**Figure 6:**
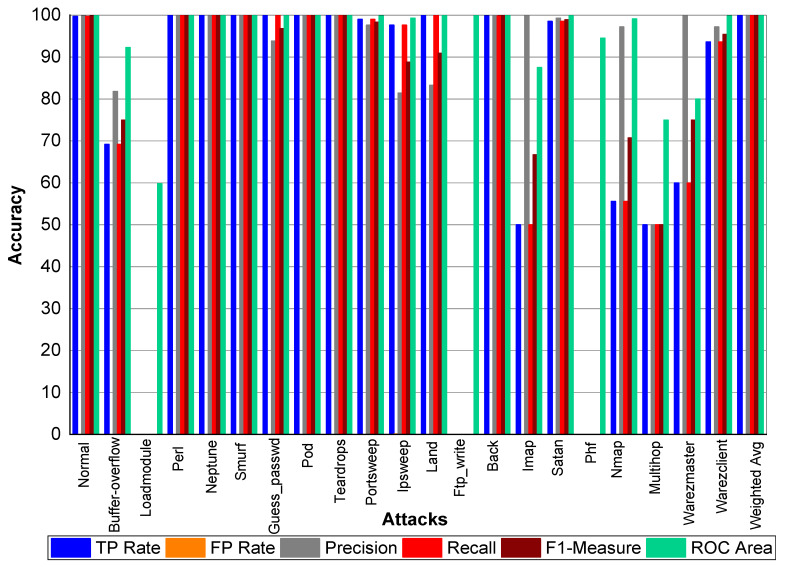
Classification report for the Bagging J48 KDD99 dataset.

**Figure 7:**
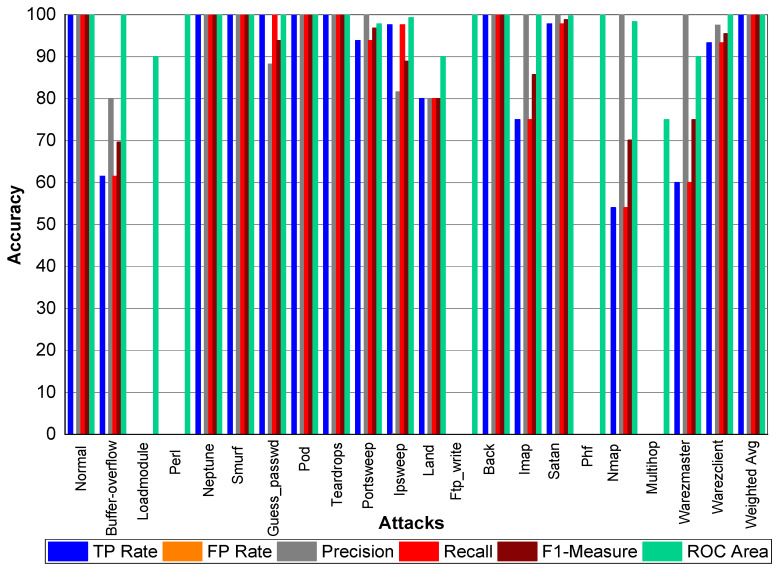
Classification report for the Bagging Random Forest KDD99 dataset.

From Table 23 and Figure 5, we can conclude that Normal class achieved 99.80% precision, 99.70% recall, and 99.80% F1-Measure, respectively. Loadmodule, Ftp_write, Phf, and Multihop achieved very low results. Perl, Neptune, Smurf, Guess-passwd, Pod, teardrop, and Back achieved 100% TP Rate, precision, recall, and F1-Measures, respectively. Buffer-overflow, Portsweep, Ipsweep, Land, Imap, Satan, Nmap, Warezmaster, and Warezclient also performed well and achieved on average 90% precision, recall, and F1-Measure, respectively. From Table 24 and Figure 6, we can conclude that Normal class achieved 99.80% precision, 99.70% recall, and 99.80% F1-Measure, respectively. Loadmodule, Ftp_write, and Phf achieved very low results. Perl, Neptune, Smurf, Guess-passwd, Pod, teardrop, and Land achieved 100% TP Rate, precision, recall, and F1-Measures, respectively. Buffer-overflow, Portsweep, Ipsweep, Back, Imap, Satan, Nmap, Warezmaster, and Warezclient also performed well and achieved on average 90% precision, recall, and F1-Measure, respectively. From Table 25 and Figure 7, we can conclude that Normal class achieved 99.80% precision, recall, and F1-Measure, respectively. Similarly, Buffer-overflow achieved 61.50% recall and TP Rate, 80% recall, and 69.69% F1-Measure, respectively. Loadmodule, Perl, Phf, and Multihop achieved very low results. Neptune, Smurf, Guess-passwd, Pod, teardrop, and Imap achieved 100% TP Rate, precision, recall, and F1-Measures, respectively. Buffer-overflow, Portsweep, Ipsweep, Land, Imap, Satan, Nmap, Warezmaster, and Warezclient also performed well and achieved on average 90% precision, recall and F1-Measure, respectively.

**Figure 8:**
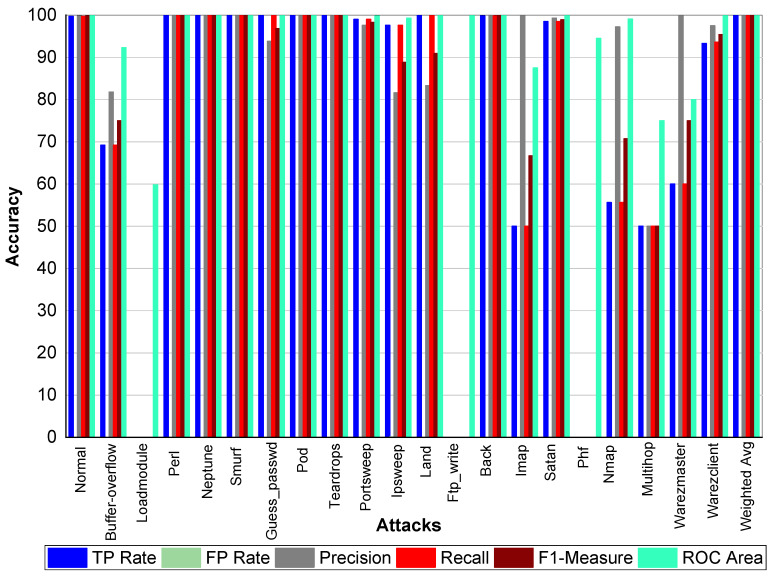
Classification report for the Bagging Reptree kdd99 dataset.

Table 26 and Figure 8 depict that Normal class achieved 99.80% precision, 99.70% recall, and 99.80% F1-Measure, respectively. Loadmodule, Ftp_write and Phf achieved very low results. Perl, Neptune, Smurf, Guess-passwd, Pod, teardrop, and Land achieved 100% TP Rate, precision, recall, and F1-Measures, respectively. Buffer-overflow, Portsweep, Ipsweep, Back, Imap, Satan, Nmap, Warezmaster, and Warezclient also performed well and achieved on average 90% precision, recall, and F1-Measure, respectively.

### 4.2. Binary Class Experiment Results for NSLKDD

Table 27 indicates that 44,481 packets are used for testing and 44,026 packets are detected correctly as normal and anomaly packets, and 455 packets were incorrectly detected; the accuracy of Adaboost J48 was 98.97%.

In Table 28, TP rate for both normal and anomaly was 99.10% and 98.90%, respectively, while FR rate for normal packets was 1.10% and, for anomaly packets, it was 0.90%, respectively. Precision, recall, and F1-Score for normal packets was 99.00%, 99.10%, and 99.00%, respectively. Similarly, for anomaly packets, the precision score was 99.00%, recall score was 98.90%, and F1-Score was 98.90%, respectively. The ROC area was 99.90%, respectively, for both normal and anomaly packets.

Table 29 indicates that 44,481 packets were used for testing and 44,072 packets were detected correctly as normal and anomaly packets, and 409 packets were incorrectly detected; the accuracy of Adaboost random forest was 99.08%. TP rate for both normal and anomaly was 99.00% and 99.20%, respectively. FR rate for normal packets was 0.8% and, for anomaly packets, it was 1.00%. Precision, recall, and F1-score for normal packets were 99.30%, 99.00%, and 99.10%, respectively. Likewise, for anomaly packets, precision score was 98.90%, recall score was 99.20%, and F1-Score was 99.00%, respectively. The ROC area was 99.80%, respectively, for both normal and anomaly packets as shown in Table 30.

Table 31 indicates that 44,481 packets were used for testing and 44,028 packets were detected correctly as normal and anomaly packets, and 453 packets were incorrectly detected; the accuracy of Adaboost reptree was 98.98%. The TP rate for both normal and anomaly was 98.70% and 99.30% respectively. The FR rate for normal packets was 0.70% and, for anomaly packets, it was 1.30%. Precision, recall, and F1-score for normal packets was 99.40%, 98.70%, and 99.00%, respectively. On the other hand, for anomaly packets, precision score was 99.30%, recall score was 99.30%, and F1-Score was 98.90%, respectively. The ROC area was 99.90%, respectively, for both normal and anomaly packets, as shown in Table 32.

Table 33 indicates that 44,481 packets were used for testing and 44,039 packets were detected correctly as normal and anomaly packets, and 442 packets were incorrectly detected; the accuracy of Bagging j48 was 99.00%. TP rate for both normal and anomaly was 99.10% and 98.90%, respectively. FR rate for normal packets was 1.10% and, for anomaly packets, it was 0.90%, respectively. Precision, recall, and F1-score for normal packets was 99.00%, 99.10%, and 99.00%, respectively. Similarly, for anomaly packets, precision score was 99.00%, recall score was 98.90%, and F1-Score was 99.00%, respectively. The ROC area was 99.90%, respectively, for both normal and anomaly packets as shown in Table 34.

Table 35 indicates that 44,481 packets were used for testing and 44,072 packets were detected correctly as normal and anomaly packets, and 409 packets were incorrectly detected; the accuracy of Bagging random forest was 99.08%. TP rate for both normal and anomaly was 99.20% and 99.10%, respectively. FR rate for normal packets was 0.90%, and, for anomaly packets, it was 0.80%, respectively. Precision, recall, and F1-Score for normal packets was 99.10%, 99.10%, and 99.10%, respectively. Similarly, for anomaly packets, precision score was 99.10%, recall score was 99.10%, and F1-Score was 99.10%, respectively. The ROC area was 99.90%, respectively, for both normal and anomaly packets, as shown in Table 36.

Table 37 indicates that 44,481 packets were used for testing, and 44,072 packets were detected correctly as normal and anomaly packets, and 409 packets were incorrectly detected; the accuracy of Bagging random forest was 99.08%. TP rate for both normal and anomaly was 99.00% and 98.90%, respectively. FR rate for normal packets was 1.10%, and, for anomaly packets, it was 1.00%, respectively. Precision, recall, and F1-score for normal packets was 99.00%, 99.00%, and 99.00%, respectively. Similarly, for anomaly packets, precision score was 98.90%, recall score was 98.90%, and F1-Score was 98.90%, respectively. The ROC area was 99.90%, respectively, for both normal and anomaly packets, as shown in Table 38.

**Figure 9:**
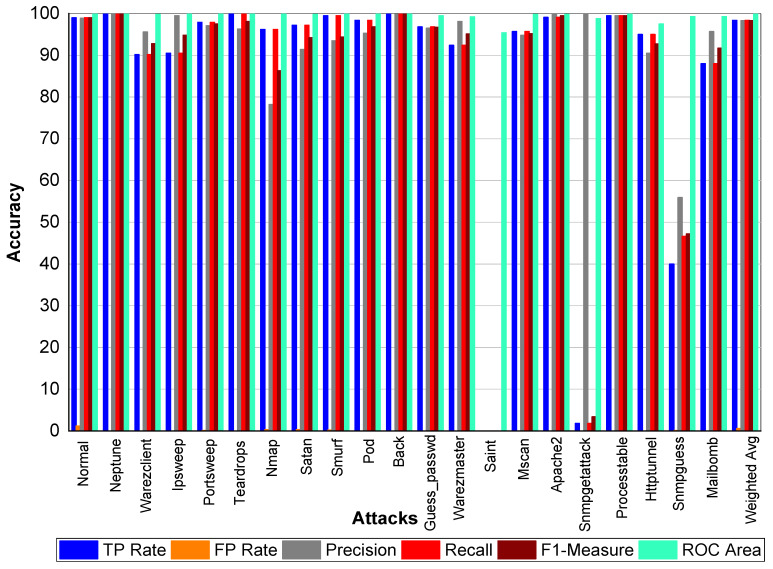
Classification report using the Adaboost J48 NSLKDD dataset.

From Table 39 and Figure 9, we can conclude that Normal class achieved 99.80% precision, recall, and F1-Measure, respectively. Neptune class achieved 99.90% precision, 100% recall, and 99.90% F1-Measure, respectively. Similarly, Warezclient achieved 95.60%, 90.20%, and 92.80% precision, recall, and F1-Measure, respectively. On the other hand, Ipsweep achieved 99.50%, 90.50%, and 94.80% precision, recall, and F1-Measure, respectively. Portsweep achieved above 97% precision, recall, and F1-Measure, respectively. Teardrop achieved 96.30%, 100%, 98.10% precision, recall, and F1-Measure, respectively. For Nmap precision, recall and F1-Measure scores were 78.20%, 96.20%, and 86.30%, respectively. Satan, Smurf, and Pod achieved on average 90% precision, recall, and F1-Measure, respectively. Back attack achieved 100% recall while 99.80% and 99.90% precision and F1-Measure, respectively. Guess_passwd achieved 96.50%, 96.80%, and 96.70% precision, recall, and F1-Measure, respectively. Saint, Snmpgetattack, and Snmpguess attack didn’t perform well. Warezmaster, Mscan, Apache 2, Processtable, Httptunnel, and Mailbomb also achieved promising results for precision, recall, F1-Measure, and for TP Rate as well.

**Figure 10:**
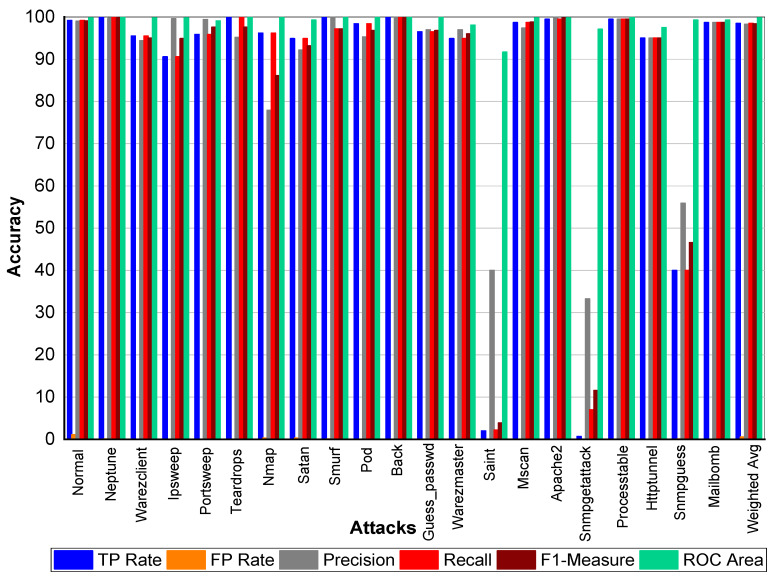
Classification report for the Adaboost Random Forest NSLKDD dataset.

From Table 40 and Figure 10, we can conclude that Normal class achieved 99.00% precision, 99.20% recall, and 99.10% F1-Measure, respectively. Neptune class achieved 99.70% precision, 100% recall, and 99.80% F1-Measure, respectively. Similarly, Warezclient achieved 94.40%, 95.50%, and 95% precision, recall, and F1-Measure, respectively. Likewise, Ipsweep achieved 99.60%, 90.60%, and 94.90% precision, recall, and F1-Measure, respectively. Portsweep achieved above 97% precision, recall, and F1-Measure, respectively. Teardrop achieved 95.20%, 100%, 97.60% precision, recall, and F1-Measure, respectively. For Nmap precision, recall, and F1-Measure scores were 77.90%, 96.20%, and 86.10%, respectively. Satan, Smurf, and Pod achieved on average 90% precision, recall, and F1-Measure, respectively. Back attack achieved 100% recall, precision, and F1-Measure, respectively. Guess_passwd achieved 97%, 96.50%, and 96.80% precision, recall, and F1-Measure, respectively. Saint, Snmpgetattack, and Snmpguess performed well. Warezmaster, Mscan, Apache2, Processtable, Httptunnel, and Mailbomb also achieved promising results for precision, Recall, F1-Measure, and for TP Rate as well.

**Figure 11:**
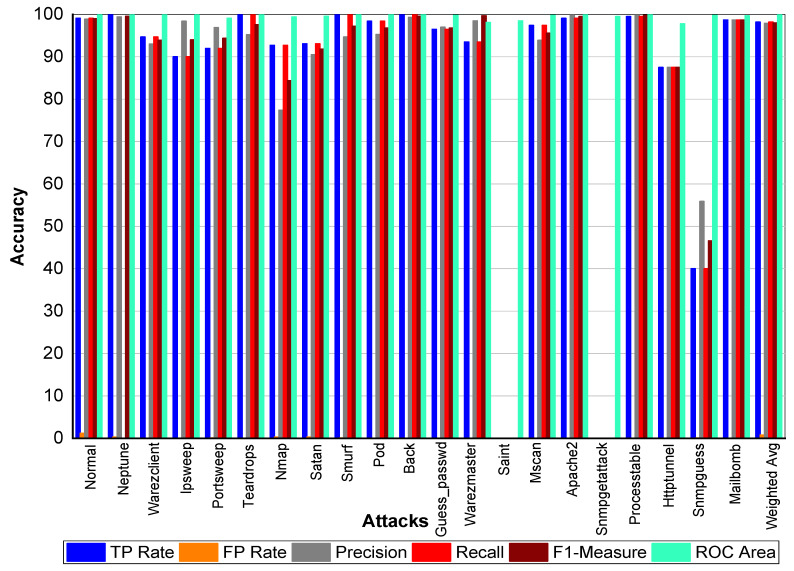
Classification report for the Adaboost Reptree NSLKDD dataset.

From Table 41 and Figure 11, we can conclude that Normal class achieved 98.90% precision, 99.10% recall, and 99% F1-Measure, respectively. Neptune class achieved 99.40% precision, 99.90% recall, and 99.60% F1-Measure, respectively. Similarly, Warezclient achieved 93%, 94.70%, and 93.90% precision, recall, and F1-Measure, respectively. In addition, Ipsweep achieved 98.40%, 90%, and 94% precision, recall, and F1-Measure, respectively. Portsweep achieved above 96.90% precision, 92% recall, and 94.40% F1-Measure, respectively. Teardrop achieved 95.20%, 100%, 97.60% precision, recall, and F1-Measure, respectively. For Nmap precision, recall and F1-Measure scores were 74.40%, 92.70%, and 84.40%, respectively. Satan, Smurf, and Pod achieved on average 93% precision, recall, and F1-Measure, respectively. Back attack achieved 100% recall while 99.30% and 99.60% precision, and F1-Measure, respectively. Guess_passwd achieved 97%, 96.50% and 96.80% precision, recall, and F1-Measure, respectively. Saint, Snmpgetattack, and Snmpguess performed well. Warezmaster, Mscan, Apache2, Processtable, Httptunnel, and Mailbomb also achieved promising results for precision, recall, and F1-Measure.

**Figure 12:**
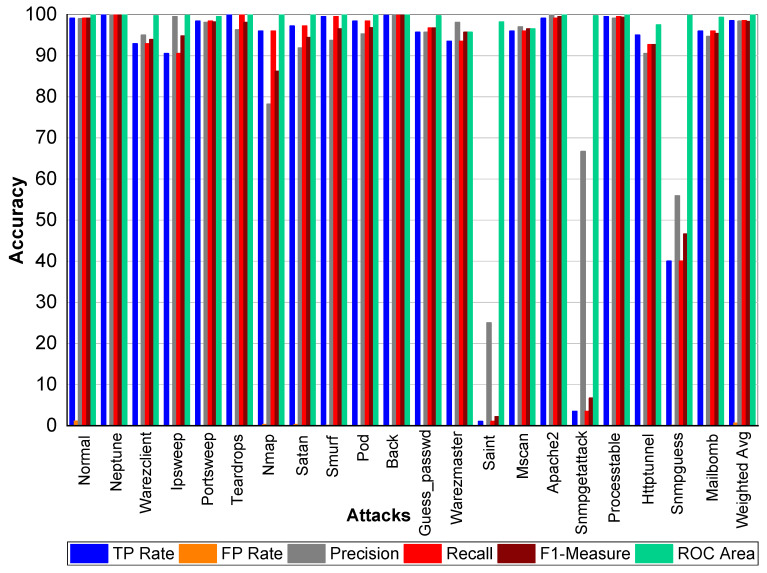
Classification report for the Bagging J48 NSLKDD dataset.

From Table 42 and Figure 12, we can conclude that Normal class achieved 99% Precision, 99.10% Recall, and 99.10% F1-Measure, respectively. Neptune class achieved 99.90% Precision, 100% Recall, and 99.90% F1-Measure, respectively. Similarly, Warezclient achieved 95%, 992%, and 93% Precision, Recall, and F1-Measure, respectively. Meanwhile, Ipsweep achieved 99%, 90%, and 94% Precision, Recall, and F1-Measure, respectively. Portsweep achieved above 98.10% precision, 98.40% Recall, and 98.20% F1-Measure, respectively. Teardrop achieved 96.30%, 100%, 98.60% Precision, Recall, and F1-Measure, respectively. For Nmap Precision, Recall, and F1-Measure scores are 78%, 96%, and 86%, respectively. In addition, 91%, 97%, and 94% Precision, Recall, and F1-Measure are achieved for Satan attack. Smurf and Pod achieved on average 96% Precision, Recall, and F1-Measure, respectively. Back attack achieved 100% Recall while 99.30% and 99.60% Precision, and F1-Measure, respectively. Guess_passwd achieved 97%, 96.50%, and 96.80% Precision, Recall, and F1-Measure, respectively. Saint, Snmpgetattack, and Snmpguess attacks did not perform well. Warezmaster, Mscan, Apache2, Processtable, Httptunnel, and Mailbomb also achieved promising results for precision, Recall, and F1-Measure.

**Figure 13:**
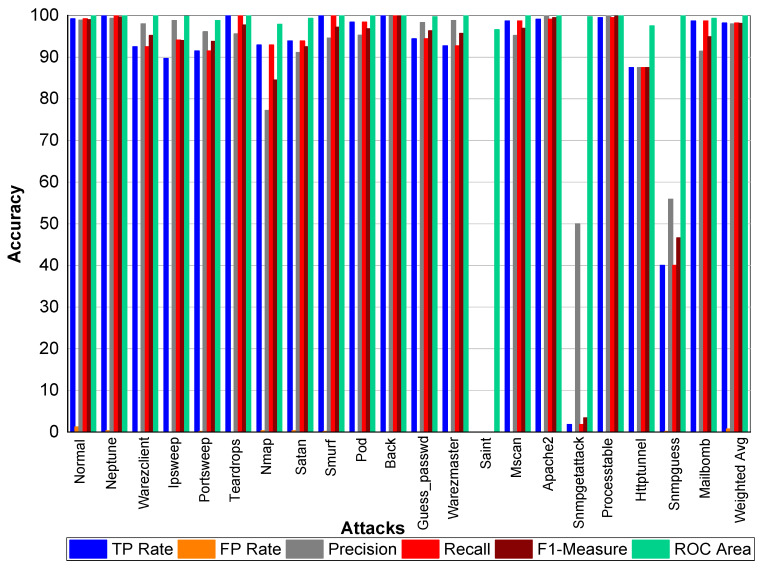
Classification report for the Bagging Random Forest NSLKDD dataset.

From Table 43 and Figure 13, we can conclude that Normal class achieved 99.10% Precision, 99.20% Recall, and 99.20% F1-Measure, respectively. Neptune class achieved 99.80% Precision, 100% Recall, and 99.90% F1-Measure, respectively. In addition, Warezclient achieved 93%, 98.90%, and 96% Precision, Recall, and F1-Measure, respectively. Likewise, Ipsweep achieved 99.70%, 90.90%, and 95.10% Precision, Recall, and F1-Measure, respectively. Portsweep achieved above 99% precision, 96% Recall, and 97% F1-Measure, respectively. Teardrop achieved 96.30%, 99.60%, 97.90% Precision, Recall, and F1-Measure, respectively. For Nmap Precision, Recall, and F1-Measure scores are 78.60%, 95.30% and 86.20%, respectively. In addition, 91.90%, 96.70%, 94.20% Precision, Recall, and F1-Measure are achieved for Satan attack. Smurf achieved 94%, 99%, and 97% Precision, Recall, and F1-Measure, respectively. Pod achieved on average 96% Precision, Recall, and F1-Measure, respectively. Back attack achieved 100% Precision, Recall, and F1-Measure, respectively. Guess_passwd achieved 97%, 96.50%, and 96.80% Precision, Recall, and F1-Measure, respectively. Saint, Snmpgetattack, and Snmpguess performed well. Warezmaster, Mscan, Apache2, Processtable, Httptunnel, and Mailbomb also achieved promising results for precision, Recall, and F1-Measure. All the attacks achieved above 90% results for all the evaluation matrixs.

**Figure 14:**
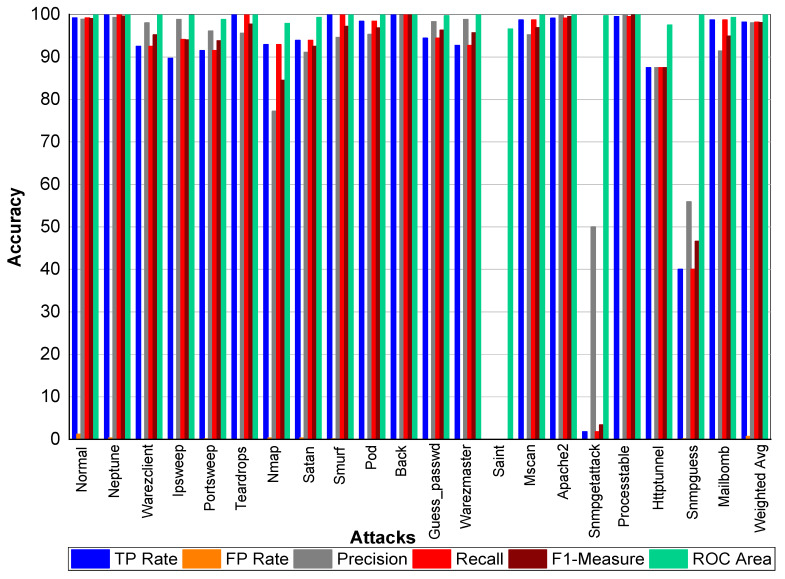
Classification report for the Reptree NSLKDD dataset.

From Table 44 and Figure 14, we depict that Normal class achieved 98% precision, 99.20% recall, and 99.00% F1-Measure, respectively. Neptune class achieved 99.30% precision, 99.90% recall, and 99.60% F1-Measure, respectively, while Warezclient achieved 98%, 92%, and 95% precision, recall, and F1-Measure, respectively. Similarly, Ipsweep achieved 98%, 94%, and 94% precision, recall, and F1-Measure, respectively. Portsweep achieved above 96% precision, 91% recall, and 93% F1-Measure, respectively. Teardrop achieved 95.60%, 100%, 97.70% precision, recall, and F1-Measure, respectively. For Nmap precision, recall, and F1-Measure scores are 77%, 92%, and 84%, respectively. In addition, 91%, 93%, and 92% precision, recall, and F1-Measure were achieved for Satan attack. Smurf achieved 94%, 99%, and 97% precision, recall, and F1-Measure, respectively. Pod achieved on average 97% precision, recall, and F1-Measure, respectively. Back attack achieved 99.80% precision, 100% recall, and 99.90% F1-Measure, respectively. Guess_passwd achieved 98%, 94%, and 96% precision, recall, and F1-Measure, respectively. Saint, Snmpgetattack, and Snmpguess performed well. Warezmaster, Mscan, Apache2, Processtable, Httptunnel, and Mailbomb also achieved promising results for precision, recall, and F1-Measure. These attacks achieved above 95% results for all the evaluation matrixs.

## 5. Discussion

In this section, we will discuss our key outcomes as well as comparison with previous work. Therefore, Table 45, Table 46 and Table 47 provide the detailed results of our whole work. Hence, we will discuss them one by one in detail.

From Table 45, we conclude that, with base machine learning classifier j48, random forest, and Reptree, we used Adaboost and Bagging to make predictions more accurate on KDD99 and NSLKDD datasets, for binary and multi classes. J48, Random Forest, and Reptree with Adaboost achieved 99.90 true positive (TP) rate and 00.00% false positive (FP) rate, respectively. Meanwhile, precision recall and F1-score were 99.90%, respectively, for all base classifiers with Adaboost and Bagging, respectively, using the KDD99 dataset. On the NSLKDD dataset, true positive and false positive scores were 98.40% and 00.60%, respectively, using Adaboost with a j48 classifier. Adaboost with random forest achieved a 98.50% TP rate and 00.60% FR rate, respectively. Precision was 98.30%, recall was 98.50%, and F1-score was 98.40%, respectively. ROC area for Adaboost J48 and random forest were 99.90% and 99.80%, respectively. TP and FR rate for Adaboost Reptree was 98.20% and 00.80%, respectively. Precision was 97.90%, recall was 98.20%, and F1-score was 98.00%, respectively. TR rate for Bagging j48 was 98.50%, for Bagging random forest was 98.60%, and for Bagging reptree was 98.20%, respectively. Precision, recall, and F1-score for Bagging J48 was 98.40%, 98.50%, and 98.30%, respectively. FR rate for Bagging J48, random forest, and reptree was 00.60%, 00.50%, and 00.70%, respectively. For Bagging random forest, precision was 98.40%, and recall and F1-score were 98.60% and 98.40%, respectively. Bagging reptree achieved 98% precision, and 98.20% and 98.10% recall and F1-score, respectively.

Similarly, from Table 46, we can conclude that Adaboost and Bagging with base classifiers j48, random forest, and reptree achieved high accuracy, TR rate, precision, recall, and F1-measure and improved FP rate for both KDD99 and NSLKDD datasets. Adaboost with J48, random forest, and reptree achieved 99.30%, 99.10%, and 99.40% TP rate and 00.90%, 00.90%, and 00.70% FP rate, respectively, on the KDD99 dataset. Precision and recall scores for Adaboost J48 were both 99.30%, respectively. F1-score was 98.30% for J48 with Adaboost for multiclass. Similarly, random forest with Adaboost achieved 99.10% precision, recall, and F1-Scores, respectively. For Adaboost with reptree, we achieved 99.40% precision, recall, and F1-Score, respectively. With Bagging j48, we achieved 99.20% TP rate; likewise, for random forest and reptree, the FP rate was 99.40%, respectively. FP rate for J48 was 01.10% and 00.70%for Bagging random forest and Bagging reptree. Precision, recall, and F1-score for Bagging J48 was 99.20%, respectively. In addition, 99.40% precision, recall, and F1-score was achieved with Bagging random forest. In addition, for the nslkdd dataset using Adaboost with j48, we achieved a 99.00% TP rate and 01.00% FP rate, respectively. Furthermore, 99.10% and 00.90% TP and FP rate were achieved using Adaboost random forest. using Adaboost with reptree, we achieved 99.00% TP rate and 01.00% FP rate, respectively. Precision, recall, and F1-Score using Adaboost j48 was 99.00%, respectively. random forest with Adaboost achieved 99.10% precision, recall, and F1-Score, respectively. reptree with Adaboost achieved 99.00% precision, recall, and F1-score, respectively. In addition, 99.00%, 99.10%, and 98.90% TP rate were achieved with Bagging j48, random forest, and reptree, respectively. Furthermore, a 01.00% FP rate was achieved using j48 Bagging, 00.90% was achieved using random forest, and 01.10% was achieved with reptree. j48 with Bagging achieved 99.00% precision, recall, and F1-score, respectively. In addition, 99.10% precision, recall, and F1-score were achieved with random forest. Thus, for reptree, precision and recall were both 98.90% respectively, and F1-score was 98.10%, respectively.

In Table 47, DAR Ensemble [52] achieved 78.88% accuracy. Naive Bayes with KNN [53] achieved 82.00% Accuracy and 05.43% FP rate. Feature selection with SVM [54] achieved 82.37% detection rate and 15.00% FP rate. GAR forest with symmatrixal uncertainty [55] achieved 85.00% detection rate and 12.20% FP rate, respectively. Bagging j48 [56] achieved 82.25% detection rate and 02.79% FP rate, respectively. PCA + PSO [57] achieved 99.40% detection rate and 00.60% FP rate. Our proposed model Bagging with random forest achieved 99.90% detection rate and 00.00% FP rate, respectively, using the kdd99 dataset. Furthermore, 98.60% detection rate and 00.50% FP rate were achieved using the nslkdd dataset, which is improved compared to other state-of-the-art research.

## 6. Conclusions and Future Work

In this paper, a machine learning based intrusion detection system has been proposed. During experimentation, various ensemble machine learning algorithms have been implemented on NSLKDD and KDD99 datasets. First, NSLKDD and KDD99 datasets were collected. Then, we transformed collected data into binary classes: Attack class and Normal class. In addition, we kept them as multiple attacks (21 Attacks for both KDD99 and nslkdd datasets). At the initial stage of the experiment, various steps were included for the datasets to prepare for the experiment such as pre-processing on the datasets, min-max normalization, feature optimization, and dimensionality reduction. After best feature selection, we have applied different machine learning algorithms on both of the datasets. Ensemble random forest has outperformed all other methods in terms of accuracy, training, and false-positive rate. Experimental results prove that our method performs better in terms of detection rate, false alarm rate, and accuracy for both KDD99 and NSLKDD datasets. The FPR on the KDD99 dataset that we achieved was 0.0%, and we achieved 0.5% FPR on the NSLKDD dataset, respectively. Similarly, we achieved on average 99% testing accuracy for both KDD99 and NSLKDD datasets, respectively. The limitation of this work is that some attacks have 0 classification accuracy. The reason for this is that the data size of that attack is less than 20, while, for other attacks, data size is large. In the future, we will solve this problem using some data balancing methods like SMOTE, which balances the classes and improves the performance of lower classes as well.

## Figures and Tables

**Table 1:** KDD99 dataset binary classifications total packets.

Packets Details	Packets Count
Normal Packets	97,277
Anomaly Packets	396,731
Total Size	494,008

**Table 2:** Training and testing samples for KDD99.

Training and Testing Packets	Training and Testing Packets Count
Training Data Size	345,806
Testing Data Size	148,202

**Table 3:** Number of attacks used in this research for KDD99.

Attack Name	Category	Count
Smurf	DoS	280,790
Neptune	DoS	107,200
Normal	Normal	97,277
Back	DoS	2203
Satan	Probe	1589
Ipsweep	Probe	1247
Portsweep	Probe	1040
Warezclient	R2L	1020
Teardrop	DoS	979
Pod	DoS	264
Nmap	Probe	231
Guess passwd	R2L	53
Buffer overflow	U2R	30
Land	DoS	21
Warezmaster	R2L	20
Imap	R2L	12
Loadmodule	U2R	9
Ftp_write	R2L	8
Multihop	R2L	7
Phf	R2L	4
Perl	U2R	3

**Table 4:** NSLKDD dataset binary classifications total packets.

Packets Details	Packets Count
Normal Packets	77,054
Anomaly Packets	71,215
Total Size	148,269

**Table 5:** NSLKDD dataset binary classifications total packets.

Training and Testing Packets	Training and Testing Packets Count
Training Data Size	103,789
Testing Data Size	44,481

**Table 6:** Number of attacks used in this research for NSLKDD.

Attack Name	Count
Normal	77,054
Neptune	45,871
Satan	4368
Ipsweep	3740
Smurf	3311
Portsweep	3088
Nmap	1566
Back	1315
Guess_passwd	1284
Mscan	996
Warezmaster	964
Teardrop	904
Warezclient	890
Apache2	737
Processtable	685
Snmpguess	331
Saint	319
Mailbomb	293
Pod	242
Snmpgetattack	178
Httptunnel	133

**Table 7:** Total number of features for KDD99 and NSLKDD datasets.

S.No.	Feature Name	Feature Type	S.No.	Feature Name	Feature Type
1	Duration	Number	2	Protocol Type	Non-Numeric
3	Service	Non-Numeric	4	Flag	Non-Numeric
5	Source Bytes	Number	6	Destination Bytes	Number
7	Land	Non-Numeric	8	Wrong Fragment	Number
9	Urgent	Number	10	Hot	Number
11	Number of failed logins	Number	12	logged in	Non-Numeric
13	Number Access Files	Number	14	Root Shell	Number
15	Su_Attemped	Number	16	Number Root	Number
17	Number of File Creations	Number	18	Number Shells	Number
19	Number Access Files	Number	20	number outbound Commands	Number
21	Is Host Login	Non-Numeric	22	Is Guest Login	Non-Numeric
23	Count	Number	24	Service Count	Number
25	Serror Rate	Number	26	Service Error Rate	Number
27	Rerror Rate	Number	28	Service RError Rate	Number
29	Same Service Rate	Number	30	Different Service Rate	Number
31	Service Different Host Rate	Number	32	Dst_host_count	Number
33	Dst_host_srv_count	Number	34	Dst_host_same_srv_rate	Number
35	Dst_host_diff_srv_rate	Number	36	Dst_host_same_src_port_rate	Number
37	Dst_host_srv_diff_host_rate	Number	38	Dst_host_serror_rate	Number
39	Dst_host_srv_serror_rate	Number	40	Dst_host_rerror_rate	Number
41	Dst_host_srv_rerror_rate	Number	42	Class Label Type	Non-Numeric

**Table 8:** Number of optimal features selected using CFS.

Dataset	Selected Features Using CFS
KDD99 (For 2 Attacks)	6, 12, 23, 31, 32
KDD99 (For 21 Attacks)	2, 3, 4, 5, 6, 7, 8, 14, 23, 30, 36
nslkdd (For 2 Attacks)	1, 3, 4, 5, 7, 8, 11, 12, 13, 30, 35, 36, 37
nslkdd (For 21 Attacks)	1, 3, 4, 5, 7, 8, 11, 12, 13, 30, 35, 36, 37

**Table 9:** Confusion matrix for Adaboost J48.

	Normal	Anomaly
**Normal**	28,934	271
**Anomaly**	759	118,238

**Table 10:** Classification report for Adaboost J48.

	TP Rate	FP Rate	Precision	Recall	F1-Score	ROC Area
**Normal**	99.10	0.60	97.40	99.10	98.30	99.90
**Anomaly**	99.40	0.90	99.80	99.40	99.60	99.90

**Table 11:** Confusion matrix for the Adaboost random forest.

	Normal	Anomaly
**Normal**	28,934	271
**Anomaly**	759	118,238

**Table 12:** Classification report for the Adaboost Random Forest.

	TP Rate	FP Rate	Precision	Recall	F1-Score	ROC Area
**Normal**	99.10	0.60	97.40	99.10	98.30	99.90
**Anomaly**	99.40	0.90	99.80	99.40	99.60	99.90

**Table 13:** Confusion matrix for Adaboost Reptree.

	Normal	Anomaly
**Normal**	28,975	230
**Anomaly**	658	118,339

**Table 14:** Classification report for Ensemble Reptree.

	TP Rate	FP Rate	Precision	Recall	F1-Score	ROC Area
**Normal**	99.20	0.60	97.80	99.20	98.50	99.80
**Anomaly**	99.40	0.80	99.80	99.40	99.60	100.00

**Table 15:** Confusion matrix for Bagging J48.

	Normal	Anomaly
**Normal**	28,838	367
**Anomaly**	772	118,225

**Table 16:** Classification report for Bagging J48.

	TP Rate	FP Rate	Precision	Recall	F1 Score	ROC Area
**Normal**	98.70	0.60	97.40	98.70	98.10	99.50
**Anomaly**	99.40	1.30	99.70	99.40	99.50	100.00

**Table 17:** Confusion matrix for Bagging Random Forest.

	Normal	Anomaly
**Normal**	28,994	211
**Anomaly**	679	118,318

**Table 18:** Classification report for Bagging Random Forest.

	TP Rate	FP Rate	Precision	Recall	F1-Score	ROC Area
**Normal**	99.30	0.60	97.70	99.30	98.50	99.70
**Anomaly**	99.40	0.70	99.80	99.40	99.60	100.00

**Table 19:** Confusion matrix for Bagging Reptree.

	Normal	Anomaly
**Normal**	29,010	195
**Anomaly**	698	118,299

**Table 20:** Classification report for Bagging Reptree.

	TP Rate	FP Rate	Precision	Recall	F1-Score	ROC Area
**Normal**	99.30	0.60	97.70	99.30	98.50	99.99
**Anomaly**	99.40	0.70	99.80	99.40	99.60	100.00

**Table 21:** Multiclass classification report for KDD99 using Adaboost j48.

S.No.	Class	TP Rate	FP Rate	Precision	Recall	F1-Score	ROC Area
1	Normal	99.70	0.00	99.80	99.70	99.80	100.00
2	Buffer-overflow	46.20	0.00	100.00	46.20	63.20	99.70
3	Loadmodule	0.00	0.00	0.00	0.00	0.00	99.10
4	Perl	100.00	0.00	33.33	100.00	50.00	100.00
5	Neptune	100.00	0.00	100.00	100.00	100.00	100.00
6	Smurf	100.00	0.00	100.00	100.00	100.00	100.00
7	Guess_passwd	100.00	0.00	93.80	100.00	96.80	100.00
8	Pod	100.00	0.00	100.00	100.00	100.00	100.00
9	Teardrop	100.00	0.00	100.00	100.00	100.00	100.00
10	Portsweep	99.30	0.00	95.30	99.30	97.30	100.00
11	Ipsweep	97.90	0.10	81.60	99.90	89.00	99.20
12	Land	100.00	0.00	83.30	100.00	90.90	100.00
13	Ftp_write	0.00	0.00	0.00	0.00	0.00	100.00
14	Back	99.70	0.00	99.80	99.70	99.80	100.00
15	Imap	50.00	0.00	100.00	50.00	66.70	100.00
16	Satan	98.50	0.00	99.10	98.50	98.80	100.00
17	Phf	0.00	0.00	0.00	0.00	0.00	100.00
18	Nmap	55.60	0.00	97.20	55.60	70.70	99.80
19	Multihop	50.00	0.00	33.33	50.00	40.00	81.70
20	Warezmaster	60.00	0.00	100.00	60.00	75.00	71.70
21	Warezclient	93.00	0.00	97.10	93.00	95.00	99.10
22	Weighted Avg	99.90	0.00	99.90	99.90	99.90	100.00

**Table 22:** Multiclass classification report for KDD99 using Adaboost Random Forest.

S.No.	Class	TP Rate	FP Rate	Precision	Recall	F1-Score	ROC Area
1	Normal	99.80	0.00	99.90	99.80	99.80	100.00
2	Buffer-overflow	61.50	0.00	88.90	61.50	72.70	96.10
3	Loadmodule	20.00	0.00	33.33	20.00	25.00	89.70
4	Perl	100.00	0.00	100.00	100.00	100.00	100.00
5	Neptune	100.00	0.00	100.00	100.00	100.00	100.00
6	Smurf	100.00	0.00	100.00	100.00	100.00	100.00
7	Guess_passwd	100.00	0.00	100.00	100.00	100.00	100.00
8	Pod	100.00	0.00	100.00	100.00	100.00	100.00
9	Teardrop	100.00	0.00	100.00	100.00	100.00	100.00
10	Portsweep	96.90	0.00	99.30	96.90	98.10	100.00
11	Ipsweep	97.90	0.10	81.60	97.90	89.00	99.40
12	Land	80.00	0.00	80.00	80.00	80.00	99.90
13	Ftp_write	0.00	0.00	0.00	0.00	0.00	100.00
14	Back	100.00	0.00	100.00	100.00	100.00	100.00
15	Imap	100.00	0.00	100.00	100.00	100.00	100.00
16	Satan	98.70	0.00	99.40	98.70	99.00	99.90
17	Phf	100.00	0.00	100.00	100.00	100.00	100.00
18	Nmap	52.40	0.00	100.00	52.40	68.80	99.10
19	Multihop	50.00	0.00	100.00	50.00	66.70	100.00
20	Warezmaster	60.00	0.00	75.00	60.00	66.70	99.80
21	Warezclient	94.50	0.00	97.80	94.50	96.10	98.80
22	Weighted Avg	99.90	0.00	99.90	99.90	99.90	100.00

**Table 23:** Multiclass classification report for KDD99 using Adaboost Reptree.

S.No.	Class	TP Rate	FP Rate	Precision	Recall	F1-Score	ROC Area
1	Normal	99.70	0.00	99.80	99.70	99.80	100.00
2	Buffer-overflow	53.80	0.00	77.80	53.80	63.60	99.90
3	Loadmodule	0.00	0.00	0.00	0.00	0.00	99.90
4	Perl	0.00	0.00	0.00	0.00	0.00	100.00
5	Neptune	100.00	0.00	99.90	100.00	100.00	100.00
6	Smurf	100.00	0.00	100.00	100.00	100.00	100.00
7	Guess_passwd	100.00	0.00	83.30	100.00	90.90	100.00
8	Pod	100.00	0.00	100.00	100.00	100.00	100.00
9	Teardrop	100.00	0.00	100.00	100.00	100.00	100.00
10	Portsweep	94.80	0.00	99.30	94.80	97.00	99.40
11	Ipsweep	97.10	0.10	81.30	97.10	88.50	99.90
12	Land	80.00	0.00	80.00	80.00	80.00	99.90
13	Ftp_write	0.00	0.00	0.00	0.00	0.00	100.00
14	Back	100.00	0.00	99.80	100.00	99.90	100.00
15	Imap	75.00	0.00	100.00	75.00	85.70	85.10
16	Satan	98.50	0.00	98.50	98.50	98.50	99.90
17	Phf	0.00	0.00	0.00	0.00	0.00	99.30
18	Nmap	52.40	0.00	100.00	52.40	68.80	99.90
19	Multihop	0.00	0.00	0.00	0.00	0.00	99.80
20	Warezmaster	60.00	0.00	100.00	60.00	75.00	88.00
21	Warezclient	93.30	0.00	97.50	93.30	95.30	100.00
22	Weighted Avg	99.90	0.00	99.90	99.90	99.90	100.00

**Table 24:** Multiclass classification report for KDD99 using Bagging J48.

S.No.	Class	TP Rate	FP Rate	Precision	Recall	F1-Score	ROC Area
1	Normal	99.70	0.00	99.80	99.70	99.80	100.00
2	Buffer-overflow	69.20	0.00	81.80	69.20	75.00	92.30
3	Loadmodule	0.00	0.00	0.00	0.00	0.00	59.80
4	Perl	100.00	0.00	100.00	100.00	100.00	100.00
5	Neptune	100.00	0.00	100.00	100.00	100.00	100.00
6	Smurf	100.00	0.00	100.00	100.00	100.00	100.00
7	Guess_passwd	100.00	0.00	93.80	100.00	96.80	100.00
8	Pod	100.00	0.00	100.00	100.00	100.00	100.00
9	Teardrop	100.00	0.00	100.00	100.00	100.00	100.00
10	Portsweep	99.00	0.00	97.60	99.00	98.30	99.80
11	Ipsweep	97.60	0.10	81.40	97.60	88.80	99.30
12	Land	100.00	0.00	83.30	100.00	90.90	100.00
13	Ftp_write	0.00	0.00	0.00	0.00	0.00	99.90
14	Back	99.80	0.00	100.00	99.80	99.90	100.00
15	Imap	50.00	0.00	100.00	50.00	66.70	87.50
16	Satan	98.50	0.00	99.30	98.50	98.90	99.90
17	Phf	0.00	0.00	0.00	0.00	0.00	94.50
18	Nmap	55.60	0.00	97.20	55.60	70.70	99.10
19	Multihop	50.00	0.00	50.00	50.00	50.00	75.00
20	Warezmaster	60.00	0.00	100.00	60.00	75.00	80.00
21	Warezclient	93.60	0.00	97.20	93.60	95.40	99.80
22	Weighted Avg	99.90	0.00	99.90	99.90	99.90	100.00

**Table 25:** Multiclass classification report for KDD99 using the Bagging Random Forest.

S.No.	Class	TP Rate	FP Rate	Precision	Recall	F1-Score	ROC Area
1	Normal	99.80	0.00	99.80	99.80	99.80	100.00
2	Buffer-overflow	61.50	0.00	80.00	61.50	69.69	100.00
3	Loadmodule	0.00	0.00	0.00	0.00	0.00	90.00
4	Perl	0.00	0.00	0.00	0.00	0.00	100.00
5	Neptune	100.00	0.00	99.90	100.00	99.90	100.00
6	Smurf	100.00	0.00	100.00	100.00	100.00	100.00
7	Guess_passwd	100.00	0.00	88.20	100.00	93.80	100.00
8	Pod	100.00	0.00	100.00	100.00	100.00	100.00
9	Teardrop	100.00	0.00	100.00	100.00	100.00	100.00
10	Portsweep	93.80	0.00	100.00	93.80	96.80	97.80
11	Ipsweep	97.60	0.10	81.60	97.60	88.90	99.30
12	Land	80.00	0.00	80.00	80.00	80.00	90.00
13	Ftp_write	0.00	0.00	0.00	0.00	0.00	100.00
14	Back	100.00	0.00	99.80	100.00	99.90	100.00
15	Imap	75.00	0.00	100.00	75.00	85.70	100.00
16	Satan	97.80	0.00	99.80	97.80	98.80	99.70
17	Phf	0.00	0.00	0.00	0.00	0.00	100.00
18	Nmap	54.00	0.00	100.00	54.00	70.10	98.30
19	Multihop	0.00	0.00	0.00	0.00	0.00	75.00
20	Warezmaster	60.00	0.00	100.00	60.00	75.00	90.00
21	Warezclient	93.30	0.00	97.50	93.30	95.43	100.00
22	Weighted Avg	99.90	0.00	99.90	99.90	99.90	100.00

**Table 26:** Multiclass classification report for KDD99 using Bagging Reptree.

S.No.	Class	TP Rate	FP Rate	Precision	Recall	F1-Score	ROC Area
1	Normal	99.70	0.00	99.80	99.70	99.80	100.00
2	Buffer-overflow	69.20	0.00	81.80	69.20	75.00	92.30
3	Loadmodule	0.00	0.00	0.00	0.00	0.00	59.80
4	Perl	100.00	0.00	100.00	100.00	100.00	100.00
5	Neptune	100.00	0.00	100.00	100.00	100.00	100.00
6	Smurf	100.00	0.00	100.00	100.00	100.00	100.00
7	Guess_passwd	100.00	0.00	93.80	100.00	96.80	100.00
8	Pod	100.00	0.00	100.00	100.00	100.00	100.00
9	Teardrop	100.00	0.00	100.00	100.00	100.00	100.00
10	Portsweep	99.00	0.00	97.60	99.00	98.30	99.80
11	Ipsweep	97.60	0.10	81.40	97.60	88.80	99.30
12	Land	100.00	0.00	83.30	100.00	90.90	100.00
13	Ftp_write	0.00	0.00	0.00	0.00	0.00	99.90
14	Back	99.80	0.00	100.00	99.80	99.90	100.00
15	Imap	50.00	0.00	100.00	50.00	66.70	87.50
16	Satan	98.50	0.00	99.30	98.50	98.90	99.90
17	Phf	0.00	0.00	0.00	0.00	0.00	94.50
18	Nmap	55.60	0.00	97.20	55.60	70.70	99.10
19	Multihop	50.00	0.00	50.00	50.00	50.00	75.00
20	Warezmaster	60.00	0.00	100.00	60.00	75.00	80.00
21	Warezclient	93.60	0.00	97.20	93.60	95.40	99.80
22	Weighted Avg	99.90	0.00	99.90	99.90	99.90	100.00

**Table 27:** Confusion matrix for Adaboost J48.

	Normal	Anomaly
**Normal**	22,944	219
**Anomaly**	236	21,082

**Table 28:** Classification report for Adaboost J48.

	TP Rate	FP Rate	Precision	Recall	F1-Score	ROC Area
**Normal**	99.10	1.10	99.00	99.10	99.00	99.90
**Anomaly**	98.90	0.90	99.00	98.90	98.90	99.90

**Table 29:** Confusion matrix for Adaboost Random Forest.

	Normal	Anomaly
**Normal**	22,920	243
**Anomaly**	116	21,152

**Table 30:** Classification report for Adaboost Random Forest.

	TP Rate	FP Rate	Precision	Recall	F1-Score	ROC Area
**Normal**	99.00	0.80	99.30	99.00	99.10	99.80
**Anomaly**	99.20	1.00	98.90	99.20	99.00	99.80

**Table 31:** Confusion matrix for Adaboost Reptree.

	Normal	Anomaly
**Normal**	22,854	309
**Anomaly**	144	21,174

**Table 32:** Classification report for Adaboost Random Forest.

	TP Rate	FP Rate	Precision	Recall	F1-Score	ROC Area
**Normal**	98.70	0.70	99.40	98.70	99.10	99.90
**Anomaly**	99.30	1.30	98.60	99.30	98.90	99.90

**Table 33:** Confusion matrix for Bagging J48.

	Normal	Anomaly
**Normal**	22,949	214
**Anomaly**	228	21,090

**Table 34:** Classification report for Bagging Random Forest.

	TP Rate	FP Rate	Precision	Recall	F1-Score	ROC Area
**Normal**	99.10	1.10	99.00	99.10	99.00	99.90
**Anomaly**	98.90	0.90	99.00	98.90	99.00	99.90

**Table 35:** Confusion matrix for Bagging Random Forest.

	Normal	Anomaly
**Normal**	22,972	191
**Anomaly**	201	21,117

**Table 36:** Classification report for Bagging Random Forest.

	TP Rate	FP Rate	Precision	Recall	F1-Score	ROC Area
**Normal**	99.20	0.90	99.10	99.20	99.20	99.90
**Anomaly**	99.10	0.80	99.10	99.10	99.10	99.90

**Table 37:** Confusion matrix for Bagging Reptree.

	Normal	Anomaly
**Normal**	22,925	238
**Anomaly**	230	21,088

**Table 38:** Classification report for Bagging Reptree.

	TP Rate	FP Rate	Precision	Recall	F1-Score	ROC Area
**Normal**	99.00	1.10	99.00	99.00	99.00	99.90
**Anomaly**	98.90	1.00	98.90	98.90	98.90	99.90

**Table 39:** Multiclass classification report for NSLKDD using Adaboost J48.

S.No.	Class	TP Rate	FP Rate	Precision	Recall	F1-Score	ROC Area
1	Normal	99.00	1.20	98.90	99.00	99.00	99.99
2	Neptune	100.00	0.00	99.90	100.00	99.90	100.00
3	Warezclient	90.20	0.00	95.60	90.20	92.80	99.80
4	Ipsweep	90.50	0.00	99.50	90.50	94.80	99.90
5	Portsweep	97.90	0.10	97.10	97.90	97.50	99.90
6	Teardrop	100.00	0.00	96.30	100.00	98.10	100.00
7	Nmap	96.20	0.30	78.20	96.20	86.30	99.90
8	Satan	97.20	0.30	91.40	97.20	94.20	99.80
9	Smurf	99.50	0.20	93.30	99.50	94.40	100.00
10	Pod	98.40	0.00	95.30	98.40	96.80	100.00
11	Back	100.00	0.00	99.80	100.00	99.90	100.00
12	Guess_passwd	96.80	0.00	96.50	96.80	96.70	99.50
13	Warezmaster	92.40	0.00	98.10	92.40	95.10	99.20
14	Saint	0.00	0.00	0.00	0.00	0.00	95.40
15	Mscan	95.70	0.00	94.80	95.70	95.20	99.80
16	Apache2	99.10	0.00	100.00	99.10	99.50	99.80
17	Snmpgetattack	1.80	0.00	100.00	1.80	3.40	98.80
18	Processtable	99.50	0.00	99.50	99.50	99.50	100.00
19	Httptunnel	95.00	0.00	90.50	95.00	92.70	97.50
20	Snmpguess	40.00	0.10	55.90	46.60	47.20	99.30
21	Mailbomb	88.00	0.00	95.70	88.00	91.70	99.30
22	Weighted Avg	98.40	0.60	98.30	98.40	98.30	99.90

**Table 40:** Multiclass classification report for NSLKDD using the Adaboost Random Forest.

S.No.	Class	TP Rate	FP Rate	Precision	Recall	F1-Score	ROC Area
1	Normal	99.20	1.10	99.00	99.20	99.10	99.80
2	Neptune	100.00	0.10	99.70	100.00	99.80	100.00
3	Warezclient	95.50	0.00	94.40	95.50	95.00	99.80
4	Ipsweep	90.60	0.00	99.60	90.60	94.90	99.80
5	Portsweep	95.90	0.00	99.40	95.90	97.60	99.10
6	Teardrop	100.00	0.00	95.20	100.00	97.60	100.00
7	Nmap	96.20	0.30	77.90	96.20	86.10	99.90
8	Satan	94.90	0.30	92.20	94.90	93.20	99.30
9	Smurf	99.90	0.10	99.90	97.20	97.20	100.00
10	Pod	98.40	0.00	95.30	98.40	96.80	100.00
11	Back	100.00	0.00	100.00	100.00	100.00	100.00
12	Guess_passwd	96.50	0.00	97.00	96.50	96.80	99.90
13	Warezmaster	94.90	0.00	97.00	94.90	96.00	98.10
14	Saint	02.00	0.00	40.00	02.20	03.90	91.70
15	Mscan	98.70	0.00	97.40	98.70	98.80	99.80
16	Apache2	99.50	0.00	100.00	99.50	99.80	99.80
17	Snmpgetattack	0.70	0.00	33.30	07.00	11.60	97.10
18	Processtable	99.50	0.00	95.00	95.00	99.00	100.00
19	Httptunnel	95.00	0.00	95.00	95.00	95.00	97.50
20	Snmpguess	40.00	0.10	55.90	40.00	46.60	99.30
21	Mailbomb	98.70	0.00	98.70	98.70	98.70	99.30
22	Weighted Avg	98.50	0.60	98.30	98.50	98.40	99.80

**Table 41:** Multiclass classification report for NSLKDD using Adaboost Reptree.

S.No.	Class	TP Rate	FP Rate	Precision	Recall	F1-Score	ROC Area
1	Normal	99.10	1.20	98.90	99.10	99.00	99.90
2	Neptune	99.90	0.30	99.40	99.90	99.60	100.00
3	Warezclient	94.70	0.00	93.00	94.70	93.90	100.00
4	Ipsweep	90.00	0.00	98.40	90.00	94.00	99.90
5	Portsweep	92.00	0.10	96.90	92.00	94.40	99.10
6	Teardrop	100.00	0.00	95.20	100.00	97.60	100.00
7	Nmap	92.70	0.30	77.40	92.70	84.40	99.40
8	Satan	93.10	0.30	90.50	93.10	91.80	99.60
9	Smurf	99.90	0.10	94.70	99.90	97.20	100.00
10	Pod	98.40	0.00	95.30	98.40	96.80	100.00
11	Back	100.00	0.00	99.30	100.00	99.60	100.00
12	Guess_passwd	96.50	0.00	97.00	96.50	96.80	99.80
13	Warezmaster	93.50	0.00	98.50	93.50	99.70	98.10
14	Saint	0.00	0.00	0.00	0.00	0.00	98.50
15	Mscan	97.40	0.00	93.90	97.40	95.60	99.80
16	Apache2	99.10	0.00	100.00	99.10	99.50	99.90
17	Snmpgetattack	0.00	0.00	0.00	0.00	0.00	99.50
18	Processtable	99.50	0.00	100.00	95.50	99.80	100.00
19	Httptunnel	87.50	0.00	87.50	87.50	87.50	97.80
20	Snmpguess	40.00	0.10	55.90	40.00	46.60	99.80
21	Mailbomb	98.70	0.00	98.70	98.70	98.70	99.70
22	Weighted Avg	98.20	0.80	97.90	98.20	98.00	99.90

**Table 42:** Multiclass classification report for NSLKDD using Bagging J48.

S.No.	Class	TP Rate	FP Rate	Precision	Recall	F1-Score	ROC Area
1	Normal	99.10	1.10	99.00	99.10	99.10	99.90
2	Neptune	100.00	0.00	99.90	100.00	99.90	100.00
3	Warezclient	92.90	0.00	95.00	92.90	93.90	99.70
4	Ipsweep	90.50	0.00	99.50	90.50	94.80	99.80
5	Portsweep	98.40	0.00	98.10	98.40	98.20	99.50
6	Teardrop	100.00	0.00	96.30	100.00	98.10	100.00
7	Nmap	96.00	0.30	78.20	96.00	86.20	99.90
8	Satan	97.20	0.30	91.90	97.20	94.40	99.90
9	Smurf	99.50	0.10	93.70	99.50	96.50	100.00
10	Pod	98.40	0.00	95.30	98.40	96.80	100.00
11	Back	99.80	0.00	99.80	99.80	99.80	100.00
12	Guess_passwd	95.70	0.00	95.70	96.70	96.70	99.70
13	Warezmaster	93.50	0.00	98.10	93.50	95.70	95.70
14	Saint	01.00	0.00	25.00	01.00	02.20	98.20
15	Mscan	96.00	0.00	97.00	96.00	96.50	96.50
16	Apache2	99.10	0.00	100.00	99.10	99.50	99.90
17	Snmpgetattack	03.50	0.00	66.70	03.50	06.70	99.70
18	Processtable	99.50	0.00	99.10	99.50	99.30	100.00
19	Httptunnel	95.00	0.00	90.50	92.70	92.70	97.50
20	Snmpguess	40.00	0.10	55.90	40.00	46.60	99.80
21	Mailbomb	96.00	0.00	94.70	96.00	95.40	99.30
22	Weighted Avg	98.50	0.60	98.40	98.50	98.30	99.90

**Table 43:** Multiclass classification report for NSLKDD using Bagging Random Forest.

S.No.	Class	TP Rate	FP Rate	Precision	Recall	F1-Score	ROC Area
1	Normal	99.20	1.10	99.10	99.20	99.20	99.90
2	Neptune	100.00	0.00	99.80	100.00	99.90	100.00
3	Warezclient	98.90	0.00	93.30	98.90	96.00	100.00
4	Ipsweep	90.90	0.00	99.70	90.90	95.10	100.00
5	Portsweep	95.50	0.00	99.20	96.50	97.90	99.80
6	Teardrop	99.60	0.00	96.30	99.60	97.90	100.00
7	Nmap	95.30	0.30	78.60	95.30	86.20	99.90
8	Satan	96.70	0.30	91.90	96.70	94.20	99.90
9	Smurf	99.90	0.10	94.50	99.90	97.70	100.00
10	Pod	98.40	0.00	95.30	98.40	96.80	100.00
11	Back	100.00	0.00	100.00	100.00	100.00	100.00
12	Guess_passwd	96.80	0.00	97.30	96.80	97.00	99.60
13	Warezmaster	94.20	0.00	98.90	94.20	96.50	99.40
14	Saint	02.00	0.00	28.60	02.00	03.80	95.10
15	Mscan	99.30	0.00	95.90	99.30	97.60	100.00
16	Apache2	99.50	0.00	100.00	99.50	99.80	100.00
17	Snmpgetattack	07.00	0.00	50.00	07.00	12.30	98.80
18	Processtable	100.00	0.00	100.00	100.00	100.00	100.00
19	Httptunnel	92.50	0.00	92.50	92.50	92.50	97.50
20	Snmpguess	40.00	0.10	55.90	40.00	46.60	99.30
21	Mailbomb	98.70	0.00	97.40	98.70	98.00	99.30
22	Weighted Avg	98.60	0.50	98.40	98.60	98.40	99.90

**Table 44:** Multiclass classification report for NSLKDD using Bagging Reptree.

S.No.	Class	TP Rate	FP Rate	Precision	Recall	F1-Score	ROC Area
1	Normal	99.20	1.20	98.90	99.20	99.00	99.90
2	Neptune	99.90	0.30	99.30	99.90	99.60	100.00
3	Warezclient	92.50	0.00	98.00	92.50	95.20	100.00
4	Ipsweep	89.70	0.00	98.80	94.10	94.00	99.80
5	Portsweep	91.50	0.10	96.10	91.50	93.80	98.80
6	Teardrop	100.00	0.00	95.60	100.00	97.70	100.00
7	Nmap	92.90	0.30	77.20	92.90	84.50	97.90
8	Satan	93.90	0.30	91.10	93.90	92.50	99.30
9	Smurf	98.40	0.00	94.60	99.90	97.20	100.00
10	Pod	98.40	0.00	95.30	98.40	96.80	100.00
11	Back	100.00	0.00	99.80	100.00	99.90	100.00
12	Guess_passwd	94.40	0.00	98.30	94.40	96.30	99.70
13	Warezmaster	92.70	0.00	98.80	92.70	95.70	100.00
14	Saint	0.00	0.00	0.00	0.00	0.00	96.60
15	Mscan	98.70	0.00	95.20	98.70	96.90	100.00
16	Apache2	99.10	0.00	100.00	99.10	99.50	99.90
17	Snmpgetattack	01.80	0.00	50.00	01.80	03.40	99.70
18	Processtable	99.50	0.00	100.00	99.50	99.80	100.00
19	Httptunnel	87.50	0.00	87.50	87.50	87.50	97.50
20	Snmpguess	40.00	0.10	55.90	40.00	46.60	99.90
21	Mailbomb	98.70	0.00	91.40	98.70	94.90	99.30
22	Weighted Avg	98.20	0.70	98.00	98.20	98.10	99.90

**Table 45:** Comparison of proposed models for multiclass classification.

KDD99 Experiment Average Results							
S.No.	Proposed Models	TP Rate	FP Rate	Precision	Recall	F1-Score	ROC Area
1	Adaboost j48	99.90	0.00	99.90	99.90	99.90	100.00
2	Adaboost random forest	99.90	0.00	99.90	99.90	99.90	100.00
3	Adaboostreptree	99.90	0.00	99.90	99.90	99.90	100.00
4	Bagging j48	99.90	0.00	99.90	99.90	99.90	100.00
5	Bagging random forest	99.90	0.00	99.90	99.90	99.90	100.00
6	Bagging reptree	99.90	0.00	99.90	99.90	99.90	100.00
**NSLKDD Experiment Average Results**							
**S.No.**	**Proposed Models**	**TP Rate**	**FP Rate**	**Precision**	**Recall**	**F1-Score**	**ROC Area**
1	Adaboost j48	98.40	0.60	98.30	98.40	98.30	99.90
2	Adaboost random forest	98.50	0.60	98.30	98.50	98.40	99.80
3	Adaboostreptree	98.20	0.80	97.90	98.20	98.00	99.90
4	Bagging j48	98.50	0.60	98.40	98.50	98.30	99.90
5	Bagging random forest	98.60	0.50	98.40	98.60	98.40	99.90
6	Bagging reptree	98.20	0.70	98.00	98.20	98.10	99.90

**Table 46:** Comparison of proposed models for binary class classification.

KDD99 Experiment Average Results							
S.No.	Proposed Models	TP Rate	FP Rate	Precision	Recall	F1-Score	ROC Area
1	Adaboost j48	99.30	0.90	99.30	99.30	99.30	99.90
2	Adaboost random forest	99.10	0.90	99.10	99.10	99.10	99.80
3	Adaboostreptree	99.40	0.70	99.40	99.40	99.40	100.00
4	Bagging j48	99.20	01.10	99.20	99.20	99.20	99.90
5	Bagging random forest	99.40	0.70	99.40	99.40	99.40	99.90
6	Bagging reptree	99.40	0.70	99.40	99.40	99.40	100.00
**NSLKDD Experiment Average Results**							
**S.No.**	**Proposed Models**	**TP Rate**	**FP Rate**	**Precision**	**Recall**	**F1-Score**	**ROC Area**
1	Adaboost j48	99.00	1.00	99.00	99.00	99.00	99.90
2	Adaboost random forest	99.10	0.90	99.10	99.10	99.10	99.80
3	Adaboostreptree	99.00	1.00	99.00	99.00	99.00	99.90
4	Bagging j48	99.00	1.00	99.00	99.00	99.00	99.90
5	Bagging random forest	99.10	0.90	99.10	99.10	99.10	99.80
6	Bagging reptree	98.90	01.10	98.90	98.90	98.10	99.90

**Table 47:** Comparison analysis of our proposed model with other ensemble models.

Method	Accuracy Detection Rate (%)	FR Rate (%)
DAR Ensemble [52]	78.88	N/A
Naive Bayes-KNN-CF [53]	82.00	05.43
Feature Selection + SVM [54]	82.37	15.00
GAR Forest + Symmatrixal Uncertainity [55]	85.00	12.20
Bagging j48 [56]	84.25	02.79
PCA+PSO [57]	99.40	0.60
**Propose Model Bagging Random Forest (KDD99 dataset)**	**99.90**	**0.00**
**Propose Model Bagging Random Forest (NSLKDD dataset)**	**98.60**	**0.50**
